# Systemic silencing of *Phd2* causes reversible immune regulatory dysfunction

**DOI:** 10.1172/JCI124099

**Published:** 2019-07-29

**Authors:** Atsushi Yamamoto, Joanna Hester, Philip S. Macklin, Kento Kawai, Masateru Uchiyama, Daniel Biggs, Tammie Bishop, Katherine Bull, Xiaotong Cheng, Eleanor Cawthorne, Mathew L. Coleman, Tanya L. Crockford, Ben Davies, Lukas E. Dow, Rob Goldin, Kamil Kranc, Hiromi Kudo, Hannah Lawson, James McAuliffe, Kate Milward, Cheryl L. Scudamore, Elizabeth Soilleux, Fadi Issa, Peter J. Ratcliffe, Chris W. Pugh

**Affiliations:** 1 Nuffield Department of Medicine Research Building, Nuffield Department of Medicine, University of Oxford, Oxford, United Kingdom.; 2 Transplantation Research Immunology Group, Nuffield Department of Surgical Sciences, University of Oxford, Oxford, United Kingdom.; 3 Wellcome Trust Centre for Human Genetics, University of Oxford, Oxford, United Kingdom.; 4 Henry Wellcome Building for Molecular Physiology, Nuffield Department of Medicine, University of Oxford, Oxford, United Kingdom.; 5 Sandra and Edward Meyer Cancer Center, Weill Cornell Medicine, New York, New York, USA.; 6 Department of Cellular Pathology, Imperial College London, London, United Kingdom.; 7 MRC Centre for Regenerative Medicine, University of Edinburgh, Edinburgh, United Kingdom.; 8 Ludwig Institute for Cancer Research, Nuffield Department of Medicine, University of Oxford, Oxford, United Kingdom.; 9 Veterinary Pathology, MRC Harwell, Mary Lyon Centre, Harwell Campus, Oxford, United Kingdom.; 10 Department of Pathology, School of Biological Sciences, University of Cambridge, Cambridge, United Kingdom.; 11 The Francis Crick Institute, London, United Kingdom.

**Keywords:** Immunology, Therapeutics, Autoimmune diseases, Mouse models, hypoxia

## Abstract

Physiological effects of cellular hypoxia are sensed by prolyl hydroxylase (PHD) enzymes, which regulate HIFs. Genetic interventions on HIF/PHD pathways have revealed multiple phenotypes that extend the known biology of hypoxia. Recent studies have unexpectedly implicated HIF in aspects of multiple immune and inflammatory pathways. However, such studies are often limited by systemic lethal effects and/or use tissue-specific recombination systems, which are inherently irreversible, unphysiologically restricted, and difficult to time. To study these processes better, we developed recombinant mice that expressed tetracycline-regulated shRNAs broadly targeting the main components of the HIF/PHD pathway, permitting timed bidirectional intervention. We show that stabilization of HIF levels in adult mice through PHD2 enzyme silencing by RNA interference or inducible recombination of floxed alleles results in multilineage leukocytosis and features of autoimmunity. This phenotype was rapidly normalized on reestablishment of the hypoxia-sensing machinery when shRNA expression was discontinued. In both situations, these effects were mediated principally through the *Hif2a* isoform. Assessment of cells bearing Treg markers from these mice revealed defective function and proinflammatory effects in vivo. We believe our findings reveal a new role for the PHD2/HIF2α pathway in the reversible regulation of T cell and immune activity.

## Graphical abstract

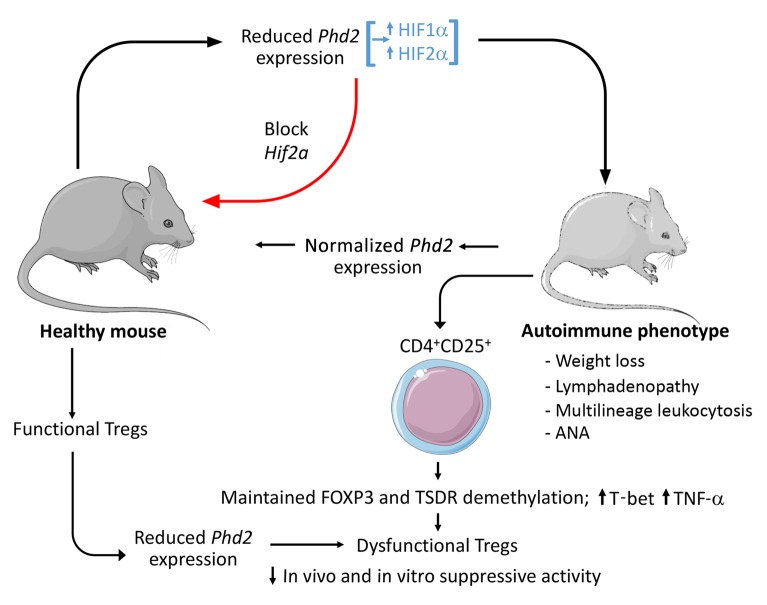

## Introduction

HIFs are transcription factors composed of 1 of 3 α chains (HIF1α, HIF2α, and HIF3α) dimerized to a β chain that mediates the transcriptional response to low oxygen tension (hypoxia). These HIF isoforms transduce different components of the hypoxia response, with HIF1α and HIF2α being the most widely expressed and best understood. In the presence of oxygen, HIFα proteins are hydroxylated by prolyl hydroxylase domain (PHD) enzymes, leading to their recognition and destruction by the von Hippel–Lindau (VHL) E3 ubiquitin ligase/proteasome pathway (1–4). Three closely related PHD enzymes have been identified: PHD1, PHD2 and PHD3, with PHD2 being the most ubiquitous and important regulator of HIF levels. Inhibition of PHD enzyme activity by low oxygen levels or other means leads to HIF activation. Together, the different HIF isoforms activate a wide range of transcriptional targets with established effects on angiogenesis, erythropoiesis, and metabolism (1–4). It is increasingly clear that the HIF pathway also influences a number of other processes less directly related to oxygen homeostasis (5, 6). Immune and inflammatory pathways are of particular interest because of their central importance in disease. HIFs are widely expressed in leukocytes and in different settings have been reported to affect cell differentiation, migration, metabolism, growth, and apoptosis (7), as well as mediate changes in barrier function (8). Oxygen levels vary across tissues in health (9), and in inflamed tissues, hypoxia is generated by a combination of reduced oxygen availability, increased metabolic demands of rapidly dividing and migrating leukocytes, and specific oxygen-consuming processes such as the respiratory (oxidative) burst (7). The effects of HIF pathway activation on immune and inflammatory pathways are therefore now of major medical importance, first, because tissue hypoxia complicates most human diseases (10) and, second, because drugs that target this pathway are now being tested in clinical trials (11).

Several studies have attempted to define physiological or pathological activities of the HIF/PHD system using genetic inactivation. At the level of the whole animal, germline disruption of *Phd2* results in developmental defects and embryonic lethality, whereas conditional knockout (KO) of *Phd2* induced systemically in adult animals leads to increased angiogenesis, erythrocytosis, and changes in energy metabolism with ultimately lethal consequences (12–15). Effects on the immune system from such global interventions have not been reported to date, perhaps because they have been obscured by these other phenotypes. In contrast, several studies have assessed the effects of targeting HIF/PHD pathway components in individual leukocyte subsets. For instance, major effects of HIF1α on innate cells, CD4^+^ and CD8^+^ T cells, Th17 cells, and Tregs have been reported (16–34). Although these experiments have undoubtedly revealed an important role of the HIF/PHD system in immune function, the results reported have not always been congruent. Differences potentially arise, because experiments that use lineage-specific, promoter-driven KO models (in which genetic changes are activated only in specific cell populations at particular stages during development and cell differentiation) may fail to capture the complexity of natural immune cell interactions and run the risk of conflating adaptive physiology with developmental effects.

We chose to investigate the HIF pathway using inducible RNA interference gene knockdown (KD) in vivo. This offers the advantages of timed and reversible specific gene silencing in mature cells, producing effects potentially more analogous to those which might occur physiologically, pathologically, or in response to pharmacological inhibition. In this study, we investigated the effects of intervention on the major HIF hydroxylase PHD2, with and without combined interventions on the 2 major HIF isoforms HIF1α and HIF2α. We show that KD of *Phd2* mRNA in mice resulted in multilineage leukocytosis and immune dysregulation with features of autoimmunity, events that were at least partially dependent on changes in the behavior of cells bearing Treg markers. This phenotype was abrogated by simultaneous KD of *Hif2a* but not *Hif1a* mRNA.

## Results

### Inducible shRNA KD.

To explore the consequences of HIF pathway component suppression on normal mouse tissues, we developed transgenic inducible shRNA mice (Supplemental Figure 1A). Ten different shRNA sequences for *Phd2* and 5 sequences each for *Hif1a* and *Hif2a* were tested initially for their effectiveness in reducing target gene mRNA levels following constitutive expression in mouse embryonic fibroblasts (MEFs) (Supplemental Table 1). For each target, we selected the 2 sequences causing the greatest percentage reduction in target gene mRNA levels for further study (*Phd2#9* and *Phd2#3*; *Hif1a#5* and *Hif1a#1*; and *Hif2a#3* and *Hif2a#1*). KH2 mouse embryonic stem (ES) cells expressing both a reverse tetracycline transactivator (*rtTA*), driven by the endogenous *GT(ROSA)26Sor* promoter *(R26-rtTA*), and a single-copy tetracycline response element (*TRE*), controlled by the *GFP-shRNA* cassette (*TRE-shRNA*) bearing the relevant shRNA sequence downstream of the collagen type I α 1 (*Col1a1*) gene, were generated using recombinase-mediated cassette exchange (RMCE) (35). Efficacy in this context was confirmed by culturing the cells on gelatin in the presence of doxycycline prior to assessment of the effects on target gene mRNA and protein levels, as well as on downstream transcription (Supplemental Table 1 and Supplemental Figure 1B). Following validation, we used ES cell clones to generate transgenic mice by blastocyst injection. Pure lines were then derived from the resultant chimeras and backcrossed with WT C57BL/6 mice for at least 6 generations prior to use.

Using these mice, we examined the consequences of sustained *Phd2* KD in adult mouse tissues by initiating *Phd2* silencing in 7- to 12-week-old mice. In order to increase the efficiency of *Phd2* silencing, we generated shPhd2#9 and shPhd2#3 mice by crossing the respective *TRE-Phd2* alleles with mice harboring a transgene encoded at the *GT(ROSA)26Sor* locus that utilizes the strong exogenous CMV early enhancer element and chicken β-actin (*CAG*) promoter to express a third-generation reverse tetracycline-regulated transactivator gene (*CAG-rtTA3*). This produces stronger and more ubiquitous target gene KD than does *R26-rtTA* (35). Following treatment with doxycycline, these inducible shPhd2#9 mice showed widespread expression of GFP translated from the targeting sequence in all organs examined when viewed at the whole tissue level (Supplemental Figure 1C). Reverse transcription PCR (RT-PCR) analysis showed that the shRNA sequence in these mice was effective at knocking down *Phd2* mRNA levels in all tissues (Supplemental Figure 1D). Although the effect varied somewhat between tissues, in all cases, the degree of *Phd2* KD was sufficient to result in upregulation of *Phd3* and *Bnip3*, two HIF target genes, but had no consistent effect on *Actb* (a gene not considered to be a HIF target) (Supplemental Figure 1D). In keeping with these results, we performed immunoblotting of liver extracts from doxycycline-treated shPhd2#9 mice, which revealed a reduction in PHD2 protein levels and stabilization of HIF1α and HIF2α proteins (Supplemental Figure 1E). When doxycycline was removed, expression of GFP disappeared, and *Phd2* mRNA levels normalized over a 2-week period (Supplemental Figure 1F). We obtained equivalent results with the shPhd2#3 mice. Overall, the shPhd2 mice we generated showed an effective but reversible KD of *Phd2* mRNA across many tissues that was sufficient to stabilize and activate HIF and induce its target genes.

### Long-term Phd2 KD results in widespread leukocytosis and immune pathology.

**Figure 1:**
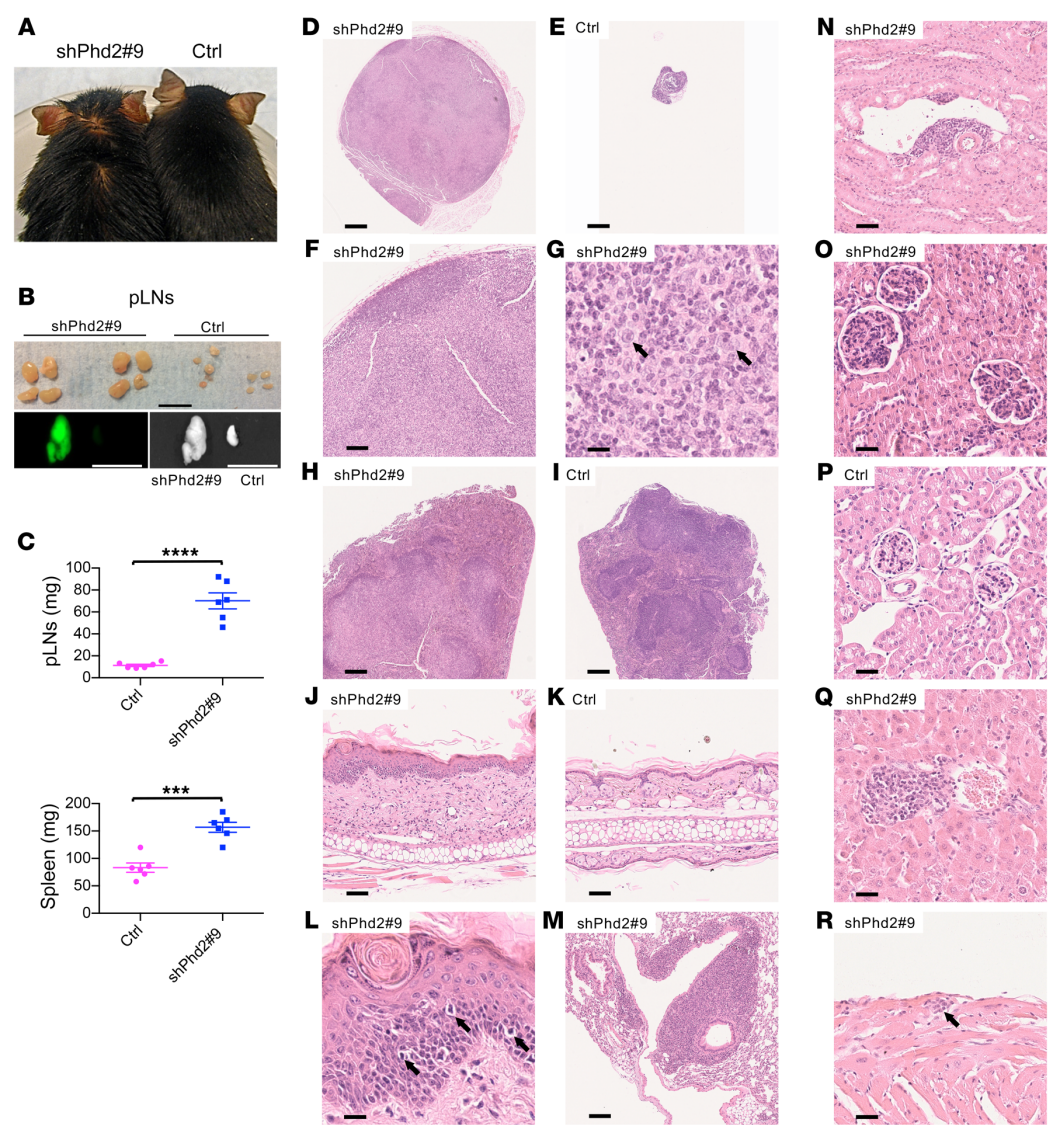
shPhd2#9 mice develop leukocyte expansion on doxycycline treatment. (**A**) Representative image of a shPhd2#9 mouse and its littermate control, both of which were treated with doxycycline (2 mg/mL with 30% sucrose drinking water ad libitum) for 4 to 5 weeks. (**B**) Representative bright-field and GFP images of pLNs from a shPhd2#9 mouse and its littermate control. Scale bars: 1 cm. (**C**) Wet weight of pLNs (4 per mouse) and spleens (1 per mouse) from shPhd2#9 mice (*n* = 6) and their littermate controls (*n* = 6). Each symbol in **C** represents an individual mouse. Data are represented as the mean ± SEM. ***P < 0.001 and ****P < 0.0001. Unpaired, independent groups of 2 were analyzed by 2-tailed Student’s *t* test. (**D**–**R**) Images of H&E-stained tissues from shPhd2#9 and littermate control mice: pLN, original magnification, ×2.5 (**D** and **E**), ×10 (**F**), and ×80 (**G**) (arrows in **G** indicate cells with oval, vesicular nuclei and eosinophilic cytoplasm); spleen, original magnification, ×5 (**H** and **I**); skin, original magnification, ×20 (**J** and **K**) and ×80 (**L**) (arrows in **L** point to exocytosis of lymphocytes into the epidermis); lung, original magnification, ×10 (**M**); kidney, original magnification, ×20 (**N**) and ×40 (**O** and **P**); liver, original magnification, ×40 (**Q**); and heart, original magnification, ×40 (**R**) (arrow in **R** indicates a focal collection of mononuclear inflammatory cells at the epicardial surface of the heart). Scale bars: 500 μm (original magnification, ×2.5, and proportionately shorter lengths at higher magnifications). Data are representative of 3 independent experiments at this time point.

On treatment for 5 to 8 weeks with doxycycline, shPhd2#9 mice became unwell, with weight loss, mild alopecia, and greasiness of the remaining hair (Figure 1A). Control mice bearing the *CAG-rtTA* but not the tetracycline-responsive element *TRE-Phd2* shRNA allele remained healthy with doxycycline treatment. We were surprised to find that shPhd2#9 mice were anemic (hematocrit 36%–38% compared with control levels of 51% to 55%) and had gross peripheral lymphadenopathy and splenomegaly (Figure 1, B and C), although the mesenteric lymph nodes (LNs) appeared macroscopically unremarkable.

Histological examination confirmed the expansion of lymphoid tissues with distortion of the normal architecture but lacking features suggestive of neoplastic transformation (Figure 1D). Eosinophilic mononuclear cells were prominent, expanding the central regions of LNs and displacing cortical areas to the extreme periphery (Figure 1, D–G), and we observed similar changes in the splenic white pulp (Figure 1, H and I). Examination of the skin of the pinna showed thickening of both the epidermis and dermis, with loss of subcutaneous fat. We observed a diffuse lymphohistiocytic infiltrate within the dermis and evidence of exocytosis of lymphocytes into the epidermis (Figure 1, J–L). Elsewhere, we detected empty hair follicles. Examination of other tissues revealed further changes, with striking perivascular accumulations of leukocytes in the bronchovascular regions of the lung (Figure 1M) and accumulations in the kidney (Figure 1N) that were similar to those seen in some human autoimmune diseases. Further examination of the kidney showed an increase in glomerular size with endocapillary hypercellularity indicative of nephritis (Figure 1, O and P); there was no tubulointerstitial inflammation. In the liver, steatosis was apparent, and we observed smaller collections of leukocytes in both periportal and perivenous sites (Figure 1Q). We also found patchy infiltration by leukocytes in the epicardium (Figure 1R) and scattered throughout skeletal muscle. Lymphoid aggregates can be seen as background changes in various organs in some mouse colonies, but in mice from this laboratory, we found that aggregates were consistently more prominent in the shPhd2#9 mice than in the control mice. Similar lymphadenopathy and splenomegaly with multilineage changes and comparable histological changes were observed with the shPhd2#3 mice that bore an independent sequence targeting *Phd2* (Supplemental Figure 2).

In a subsequent experiment, we monitored BW and hematological parameters over an 8-week period of *Phd2* KD. Differences in BW of KD mice compared with that of control mice began to be apparent after 2 weeks and were progressive (Supplemental Figure 3A). We observed the expected rise in red cell count over the first 2 weeks, but thereafter, the red cell count fell (Supplemental Figure 3B). There was a progressive rise in leukocyte counts throughout this period, and platelet counts were also higher in the shPhd2#9 mice than in the control animals (Supplemental Figure 3, C and D). Antinuclear antibodies (ANAs) could be detected in serum within the first 3 weeks of treatment.

### Immunological effects observed in shPhd2 mice are multilineage and Hif2a isoform dependent.

**Figure 2:**
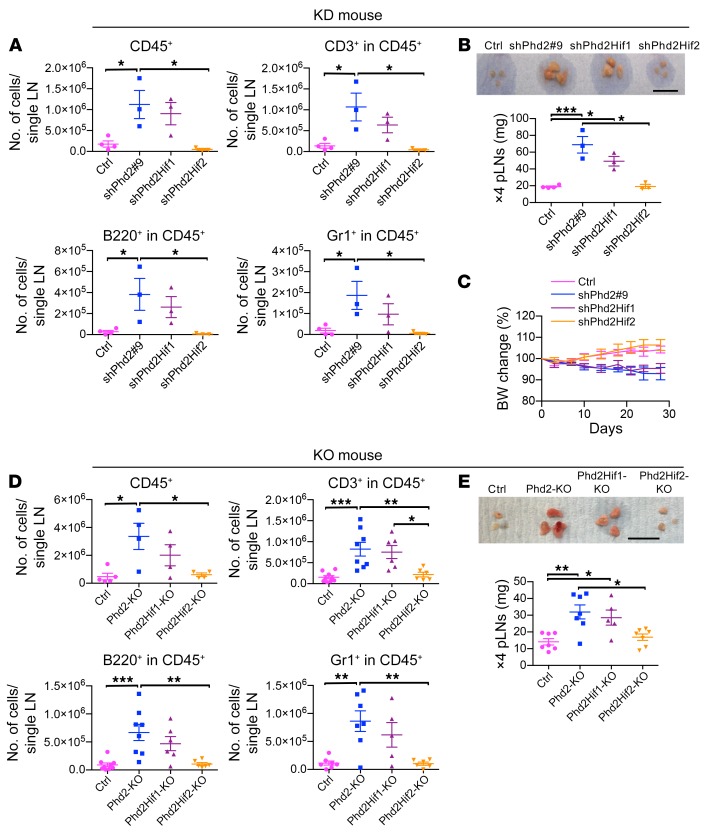
Reduction of *Hif2a* expression reverses the phenotype in inducible *Phd2*-KD and *Phd2*-KO mice. (**A**) FACS analysis of cells from pLNs of shPhd2#9 (*Phd2*-KD), shPhd2Hif1 (*Phd2*-KD/*Hif1a*-KD), shPhd2Hif2 (*Phd2*-KD/*Hif2a*-KD), and control mice for CD45, CD3, B220, and Gr1 expression. Mice were treated with doxycycline (2 mg/mL with 30% sucrose drinking water ad libitum) for 4 weeks. Data from individual mice and the mean ± SEM are represented. *P < 0.05. Groups were compared by 1-way ANOVA with Tukey’s post hoc test. (**B**) Representative bright-field images (scale bar: 1 cm) and wet weight of pLNs from the same mice. Data are represented as the mean ± SEM. *P < 0.05 and ***P < 0.001. Multigroup comparisons were analyzed by a 1-way ANOVA with Tukey’s post-hoc test. (**C**) Mean BW changes (percentage relative to day 0) of the same mice during doxycycline treatment. Data are represented as the mean ± SD (*n* = at least 3 mice per group). Groups were compared by a repeated-measures ANOVA with Tukey’s multiple comparisons post hoc test: *P* < 0.001, control versus shPhd2#9; *P* < 0.01, control versus shPhd2Hif1; NS, control versus shPhd2Hif2; NS, shPhd2#9 versus shPhd2Hif1; *P* < 0.001, shPhd2#9 versus shPhd2Hif2; *P* < 0.001, shPhd2Hif2 versus shPhd2Hif1. (**D**) FACS analysis for CD45, CD3, B220, and Gr1 expression by cells in pLNs from conditional *Phd2*-KO, Phd2Hif1-KO (*Phd2*-KO/*Hif1a*-KO), Phd2Hif2-KO (*Phd2*-KO/*Hif2a*-KO), and control mice 4 weeks after tamoxifen treatment. Data from individual mice and the mean ± SEM are represented. Groups were compared by 1-way ANOVA with Tukey’s post hoc test. *P < 0.05, **P < 0.01, and ***P < 0.001. (**E**) Representative bright-field images (scale bar: 1 cm) and wet weight of pLNs from the same mice. Data are represented as the mean ± SEM. *P < 0.05 and **P < 0.01. Multigroup comparisons were analyzed by a 1-way ANOVA with Tukey’s multiple comparisons post hoc test. Each symbol in **A**–**E** represents an individual mouse. Data were consistent over 3 independent experiments.

Flow cytometric analysis revealed that the lymphadenopathy was arising from effects across multiple leukocyte lineages: total leukocytes, T cells, B cells, and myeloid lineages as defined by CD45, CD3, B220, and GR1 (Ly-6G/Ly-6C) positivity (Figure 2A and Supplemental Figure 4A). We next sought to determine whether these immunological effects were present in conditional *Phd2*-KO animals, although such effects were previously unreported. We examined mice bearing a tamoxifen-inducible Cre recombinase–expressing transgene (*Rosa26Cre*^ERT2^) and that were homozygous for floxed *Phd2* alleles (*Rosa*ERT*Cre Phd2^fl/fl^*) 4 weeks after tamoxifen treatment. Peripheral lymphadenopathy was present in both the KD and KO mice (Figure 2, B and E), as was leukocyte expansion (Figure 2, A and D).

**Figure 3:**
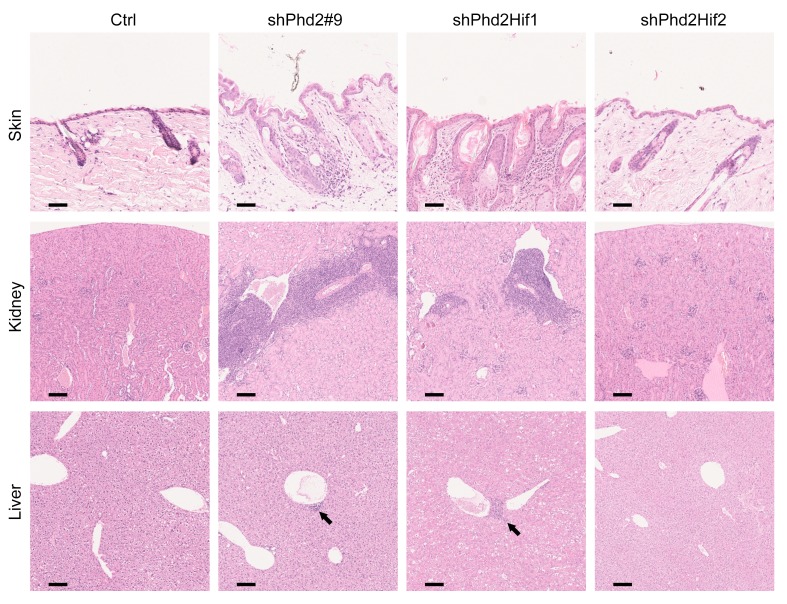
Concomitant *Hif2a* silencing ablates tissue inflammation in *Phd2*-KD mice. H&E-stained images of skin: original magnification, ×20; kidney: original magnification, ×10; and liver: original magnification, ×10 (arrows demonstrate periportal and perivenous mononuclear inflammatory cell infiltrates) from shPhd2#9 (*Phd2*-KD), shPhd2Hif1 (*Phd2*-KD/*Hif1a*-KD), shPhd2Hif2 (*Phd2*-KD/*Hif2a*-KD), and control mice treated for 4 weeks with doxycycline. Scale bars: 125 μm (original magnification, ×10, and proportionately shorter lengths at higher magnifications). Each column of images is representative of multiple sections from each organ from the same mouse. In this experiment, tissues from 2 mice of each genotype were examined.

Next, in order to assess whether the phenotype was dependent specifically on the upregulation of either HIFα isoform, we developed double-KD and double-KO mice in which both *Phd2* and *Hif1a* or *Hif2a* were targeted. shPhd2Hif1 and shPhd2Hif2 mice all carried one *CAG-rtTA3* allele, one *TRE-Phd2#9* allele, and a *TRE-Hif* allele targeting *Hif1a* or *Hif2a*, respectively. shPhd2Hif1 and shPhd2Hif2 mice were treated for 4 weeks with doxycycline at a dose of 2 mg/ml in the drinking water before analysis. The efficacy and specificity of *Hif1a* and *Hif2a* KD at the mRNA level in liver and spleen are shown in Supplemental Figure 5. Phd2Hif1-KO and Phd2Hif2-KO mice all carried one *Rosa26Cre*^ERT2^ allele and were homozygous for floxed *Phd2* alleles and homozygous for floxed alleles of *Hif1a* or *Hif2a*, respectively. Phd2Hif1-KO and Phd2Hif2-KO mice were examined 4 weeks after being treated with tamoxifen by gavage. The phenotype was reversed by concomitant Hif2a, KD, or KO in each model (Figure 2, B, C, and E). Additionally, expansion of all leukocyte lineages was completely ameliorated by concomitant HIF2α deficiency and partially reversed by concomitant HIF1α deficiency (Figure 2, A and D). Likewise, the inflammatory histological changes with *Phd2* KD were abrogated with concomitant HIF2α but not HIF1α deficiency (Figure 3).

In summary, the major features of the shPhd2 phenotype include *Hif2a*-dependent lymphadenopathy, multilineage leukocytosis, weight loss, ANA development, and immune-mediated organ pathology. These features were also seen in Phd2-KO animals.

### Reversibility of the shPhd2 phenotype.

**Figure 4:**
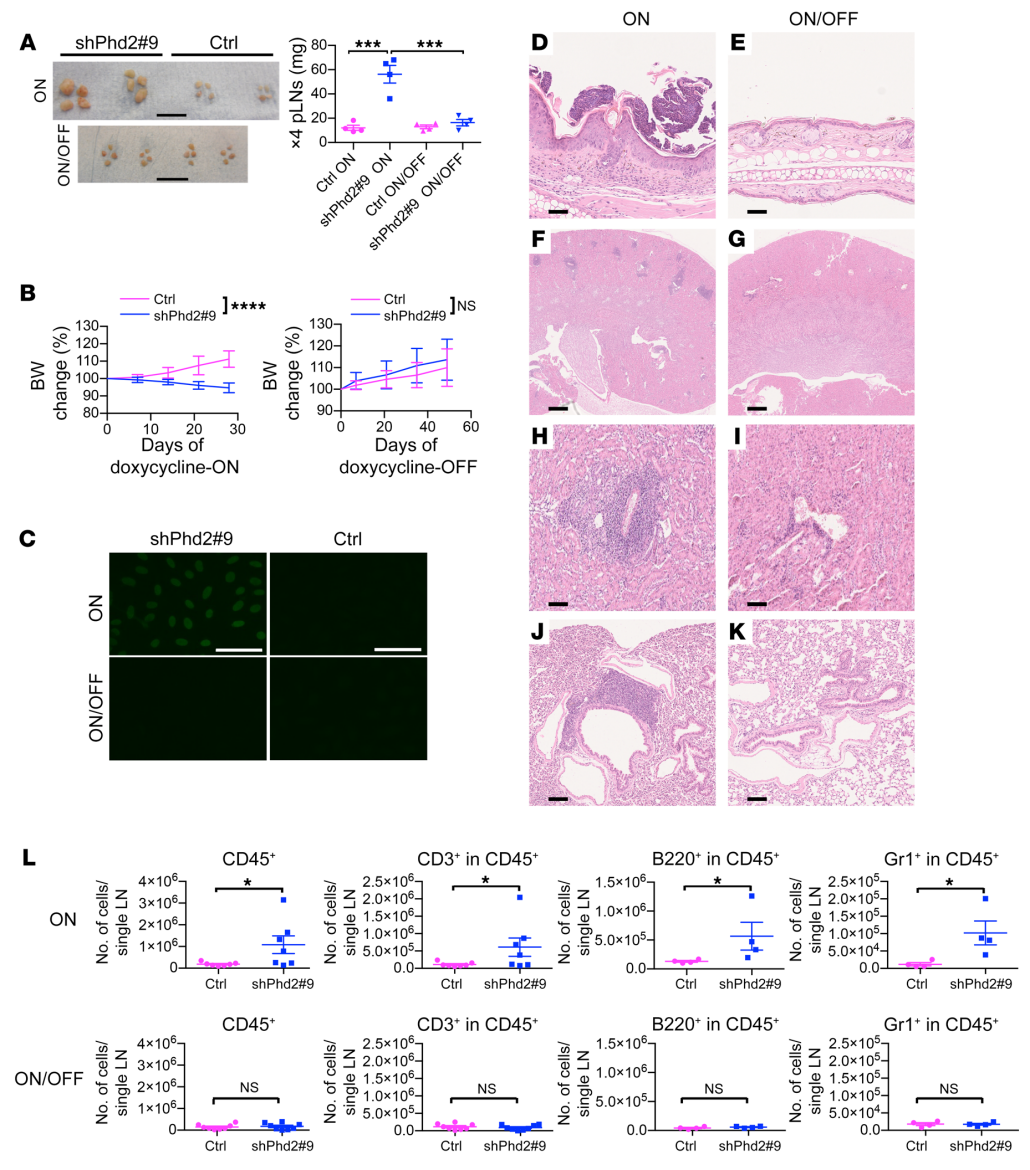
Phenotype reversal following withdrawal of doxycycline. (**A**) Representative bright-field images (scale bars: 1 cm) and wet weight of pLNs from shPhd2#9 mice and their littermate controls maintained on doxycycline for 3 to 4 weeks (doxycycline-ON group) or treated with doxycycline for 3 to 4 weeks and then analyzed 7 weeks after doxycycline withdrawal (doxycycline ON/OFF group). Data are represented as the mean ± SEM (*n* = 4/group). ***P < 0.001. Multigroup comparisons were analyzed by 1-way ANOVA with Tukey’s multiple comparisons post hoc test. (**B**) Mean percentage of BW changes of shPhd2#9 and control mice, relative to day 0 of doxycycline treatment or doxycycline withdrawal. Data are represented as the mean ± SD (*n* = 7–8/group). ****P < 0.0001, by 2-way ANOVA. (**C**) Representative fluorescence images of ANAs using mouse serum from doxycycline-ON and doxycycline-ON/OFF groups of shPhd2#9 and control mice. Scale bars: 50 μm. (**D**–**K**) H&E-stained images of (**D** and **E**) skin (original magnification, ×20); (**F**–**I**) kidney (original magnification, ×2.5 for **F** and **G** and ×10 for **H** and **I**); and (**J** and **K**) lung (original magnification, ×10) from doxycycline-ON and doxycycline-ON/OFF groups of shPhd2#9 mice. Scale bars: 500 μm (at ×2.5, and proportionately shorter lengths at higher magnifications). (**L**) FACS analysis of cells from pLNs from doxycycline-ON and doxycycline-ON/OFF groups of shPhd2#9 and control mice, analyzed for CD45, CD3, B220, and Gr1 expression. Data are represented as the mean ± SEM (*n* = at least 4/group). *P < 0.05. Unpaired, independent groups of 2 were analyzed by 2-tailed Student’s *t* test. Each symbol in **A** and **L** represents an individual mouse. Data were consistent over 2 independent experiments.

We next explored the reversibility of the observed phenotype on reexpression of *Phd2* following doxycycline withdrawal. Groups of animals were treated with doxycycline for 3 to 4 weeks until the first members of each test cohort became unwell, as described above, triggering examination of tissues and serum from a randomly selected subset of the animals. The remaining animals were taken off doxycycline, their weight was monitored, and tissues and serum were subsequently examined. The animals treated with doxycycline lost weight, developed lymphadenopathy with expansion of all lineages, and their serum became positive for ANAs, with histological examination confirming the previously described changes (Figure 4). Following doxycycline withdrawal, the lymphadenopathy regressed (Figure 4A), the mice regained weight (Figure 4B), the ANAs disappeared (Figure 4C), and the histological and immunological features improved (Figure 4, D–L). This indicates that the changes observed in shPhd2 mice were reversible and not due to either a permanent change in cell phenotype or irrevocable loss of cells with a particular function.

### The shPhd2 phenotype is transferrable by bone marrow transplantation.

**Figure 5:**
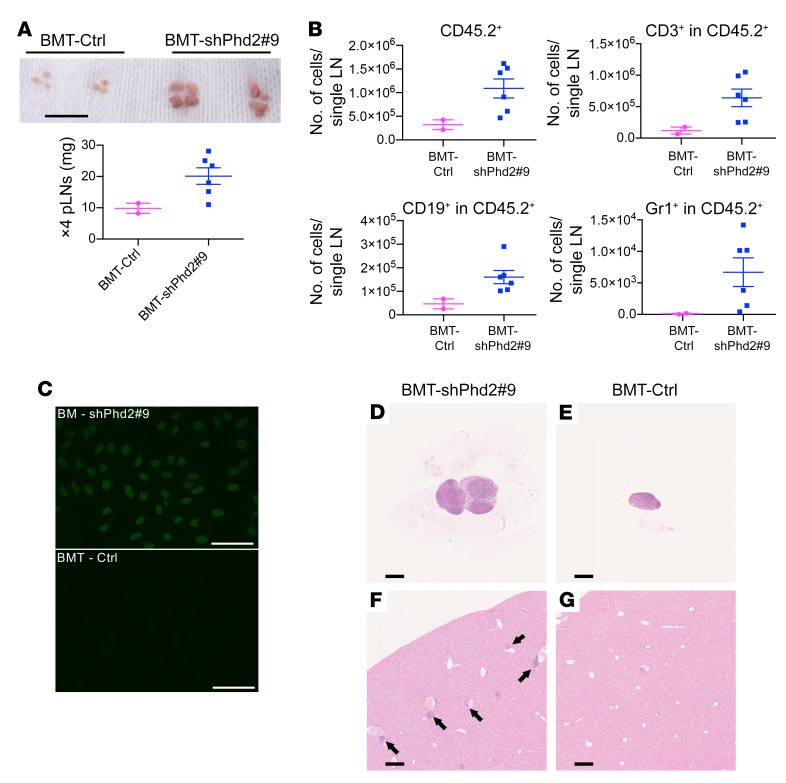
Transfer of the *Phd2*-KD phenotype by BMT. (**A**) Representative bright-field images (scale bar: 1 cm) and tissue weights of pLNs from mice that underwent BMT following 10 weeks of treatment with doxycycline (2 mg/mL with 30% sucrose drinking water ad libitum). Syngeneic congenically marked (CD45.1) mice were lethally irradiated before receiving CD45.2 shPhd2#9 BM (BMT-shPhd2#9; *n* = 6) or CD45.2 control BM (BMT-Ctrl; *n* = 2) and allowed to reconstitute for 8 weeks prior to doxycycline treatment. Data are represented as the mean ± SEM. (**B**) FACS analysis of cells in pLNs from BMT-Ctrl and BMT-shPhd2#9 mice, analyzed for CD45.2^+^, CD45.2^+^CD3^+^, CD45.2^+^CD19^+^, and CD45.2^+^Gr1^+^ expression. Data are represented as the mean ± SEM. (**C**) Representative fluorescence images of ANAs using mouse serum from BM transplant recipients. Scale bars: 50 μm. (**D**–**G**) H&E-stained images of (**D** and **E**) pLNs (original magnification, ×2.5) and (**F** and **G**) livers (original magnification, ×5) from BM transplant recipient mice. Arrows in **F** indicate inflammatory foci. Scale bars: 500 μm (original magnification, ×2.5, and proportionately shorter lengths at higher magnifications). Each symbol in **A** and **B** represents an individual mouse. The same experiment was repeated independently twice.

The phenotype observed has many features suggestive of autoimmune disease, but since the reduction of expression of *Phd2* in these mice was systemic, it was unclear whether this phenotype could arise purely through effects on the immune system. To test the role of the immune system in this phenotype, we next created bone marrow (BM) chimeras bearing shPhd2 BM in the context of an otherwise normal animal. A congenic recipient strain (CD45.1) was used to confirm complete reconstitution with donor leukocytes. Control mice, generated in parallel, received BM from mice carrying the *CAG-rtTA* allele but not the *TRE-Phd2* shRNA–targeting sequence. BM transplantation (BMT) was performed, and the transplant was allowed to engraft for 8 weeks prior to treatment with doxycycline. Upon treatment with doxycycline, mice that received KD BM (BMT-shPhd2#9), but not control BM (BMT-Ctrl), developed LN enlargement with multilineage expansion and detectable ANAs in their serum (Figure 5, A–C), although skin changes and tissue infiltration were less prominent than in the previous experiments (Figure 5, D–G). In both groups of animals, leukocytes in the peripheral blood were all of donor type (as defined by the *Ptprc*^a^ pan-leukocycte marker CD45.2) at the points of analysis. This indicates that when *Phd2* KD is restricted to the BM, it is sufficient to generate the major features of the phenotype seen in shPhd2 mice.

### Phd2 KD produces features of immune dysregulation.

**Figure 6:**
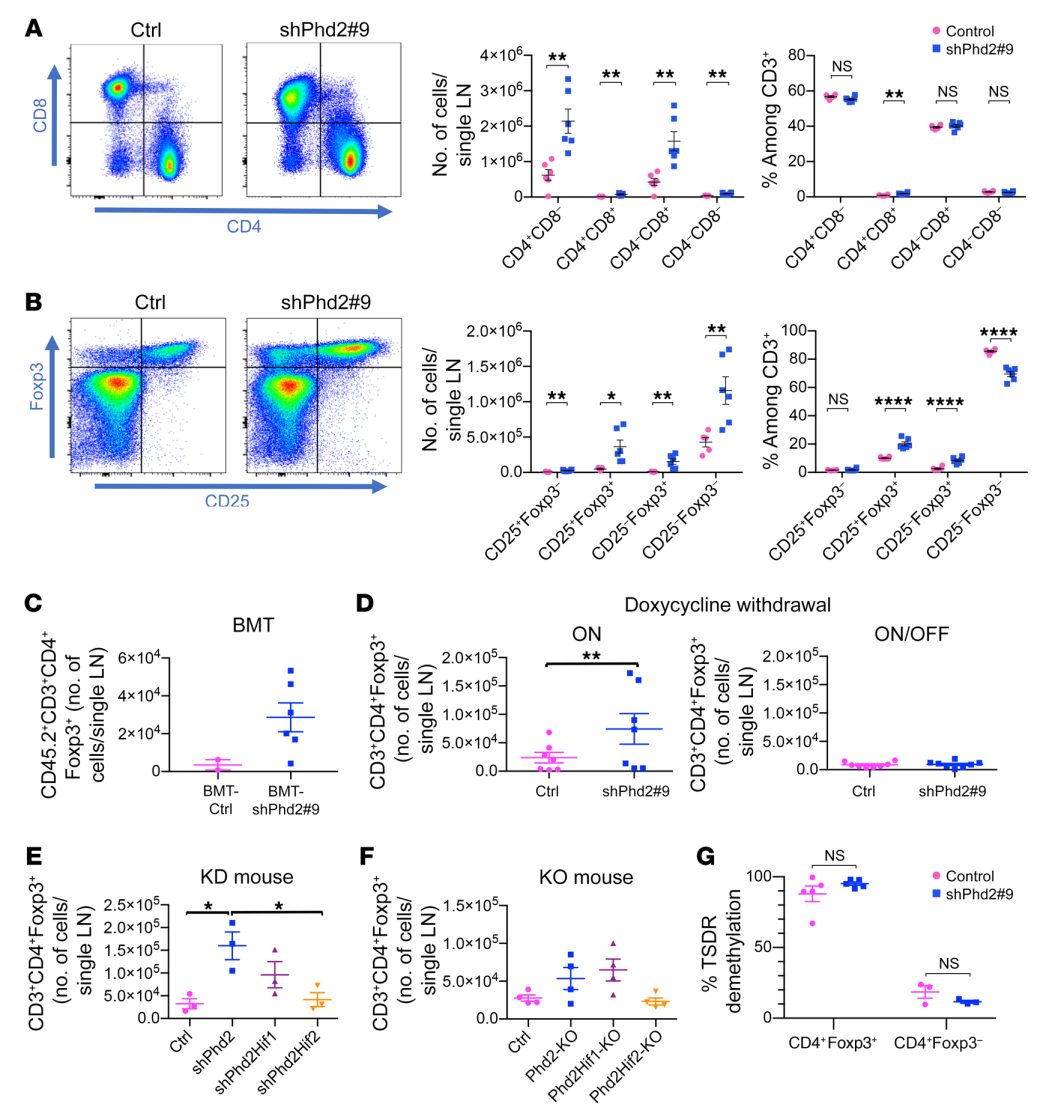
Enumeration and TSDR analysis of cells bearing helper, effector, and regulatory markers in *Phd2*-KD and -KO mice. (**A**) Gating strategy and enumeration of single-positive (CD4^+^CD8^–^ or CD4^–^CD8^+^), double-positive (CD4^+^CD8^+^), and double-negative (CD8^–^CD4^–^) cells within pLNs from control and shPhd2#9 mice following 4 weeks of doxycycline treatment (2 mg/mL with 30% sucrose drinking water ad libitum). **P < 0.01, by 2-tailed Student’s *t* test. (**B**) Expression of Foxp3 and CD25 within CD4^+^ cell populations within pLNs from control and shPhd2#9 mice following 4 weeks of doxycycline treatment. *P < 0.05, **P < 0.01, and ****P < 0.0001, by 2-tailed Student’s *t* test. (**C**) CD45.2^+^CD3^+^CD4^+^Foxp3^+^ cell numbers in pLNs from lethally irradiated syngeneic CD45.1^+^ congenically marked mice receiving BM transplants from CD45.2^+^ shPhd2#9 or CD45.2^+^ control mice. Data are represented as the mean ± SEM. (**D**) CD3^+^CD4^+^Foxp3^+^ cell numbers in the pLNs of shPhd2#9 mice and their littermate controls, treated with doxycycline for 3 to 4 weeks and analyzed directly (ON) or 7 weeks after doxycycline withdrawal (ON/OFF) (*n* = 7–8 in 2 independent assays). **P < 0.01, by 2-tailed Student’s *t* test. (**E**) CD3^+^CD4^+^Foxp3^+^ cell numbers in pLNs from shPhd2#9 (*Phd2*-KD), shPhd2Hif1 (*Phd2*-KD/*Hif1a*-KD), shPhd2Hif2 (*Phd2*-KD/*Hif2a*-KD), and control mice. *P < 0.05, by 1-way ANOVA with Tukey’s multiple comparisons post hoc test. (**F**) CD3^+^CD4^+^Foxp3^+^ cell numbers in pLNs from *Phd2*-KO, Phd2Hif1-KO (*Phd2*-KO/*Hif1a*-KO), Phd2Hif2-KO (*Phd2*-KO/*Hif2a*-KO), and control mice. Data are represented as the mean ± SEM. (**G**) Flow-sorted CD4^+^Foxp3^+^ or CD4^+^Foxp3^–^ cells from control or shPhd2#9 mice that were treated for 4 weeks with doxycycline were analyzed by prosequencing for the percentage demethylation of their TSDR on the active X chromosome. Data are represented as the mean ± SEM (*n* = at least 3/group). Unpaired, independent groups of 2 were analyzed by 2-tailed Student’s *t* test. Multigroup comparisons were analyzed by 1-way ANOVA with Tukey’s or Dunnett’s multiple comparisons post hoc test. TSDR data were analyzed by 2-way ANOVA with Sidak’s correction for multiple comparisons. Each symbol represents an individual mouse. Data in **C**–**G** are representative of 2 independent experiments.

Taken together, these features suggested that the overall phenotype in shPhd2 mice was being driven at least in part by immune dysregulation. We therefore examined these mice for the presence of cells bearing T cell subset markers related to helper, effector, and regulatory functions. Examination of the expression of CD4 and CD8 revealed an increase in the absolute numbers of CD4^+^CD8^–^ and CD4^–^CD8^+^ cells in LNs from shPhd2#9 mice compared with cells from control mice following 4 weeks of doxycycline treatment (Figure 6A and Supplemental Figure 4B). Although the absolute numbers of these cell populations changed, their representation when considered as a percentage of total CD3^+^ cells was relatively constant (Figure 6A). The absolute numbers of double-positive and double-negative cells remained low (Figure 6A and Supplemental Figure 6A). We next examined cells expressing CD25 and Foxp3, markers normally associated with Treg function, within the CD4^+^ cell population. Given the immune dysregulation in these mice, we were surprised to find that shPhd2 mice (with either targeting sequence) had increased absolute numbers of CD4^+^CD25^+^Foxp3^+^ T cells in peripheral LNs (pLNs) (Figure 6B). Foxp3^+^ cells were also more abundant in the spleen and BM, although some of these cells no longer expressed CD25 (Supplemental Figure 6, B and C). We found that the relative abundance of Foxp3^+^ cells as a proportion of CD4^+^ T cell populations was also increased (Figure 6B and Supplemental Figure 6B). We also observed increased Foxp3^+^ counts in the LNs of recipients of KD but not control BM, indicating that it was a marrow-endogenous function and not a response to nonimmune abnormalities caused by widespread PHD2 deficiency (Figure 6C). The increase in Foxp3^+^ counts was reversible with doxycycline withdrawal (Figure 6D). In keeping with the overall phenotype, the increase in the number of Foxp3^+^ cells was totally corrected by simultaneous *Hif2a* deficiency and partially reduced by *Hif1a* deficiency in the doxycycline-treated shPhd2 and *Phd2*-KO animals (Figure 6, E and F). We observed similar trends in the LNs and BM, but not the spleens, of *Phd2-*KO animals (Figure 6F and Supplemental Figure 6D). The Treg-specific demethylated region (TSDR) was demethylated in Foxp3^+^ cells isolated from doxycycline-treated shPhd2 mice (Figure 6G).

**Figure 7:**
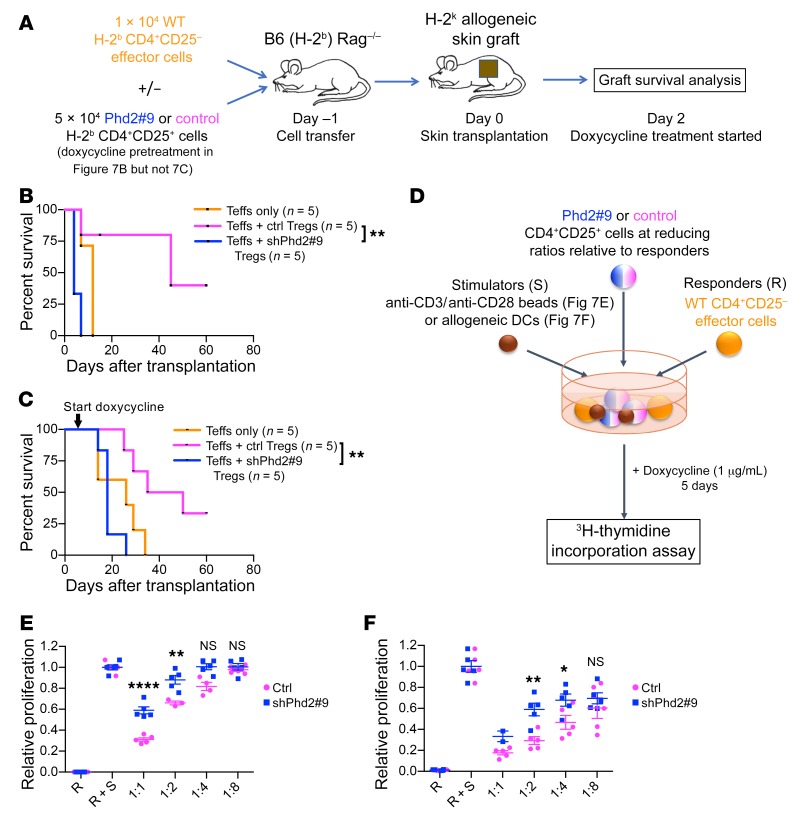
Treg functional assays. (**A**) Schematic of in vivo experimental plan. C57BL/6 *Rag1^–/–^* (H-2^b^) mice received CD4^+^CD25^–^ Teffs with or without H-2^b^ CD4^+^CD25^+^ cells from shPhd2#9 or control mice that had received either 1 week of doxycycline (2 mg/ml in drinking water, ad libitum) pretreatment or no doxycycline pretreatment. One day later, mice received an allogeneic H-2^k^ skin graft. Mice receiving cells from doxycycline-treated donors remained on doxycycline (**B**), whereas those receiving cells from untreated donors started doxycycline treatment from day 2 after transplantation (**C**). Allograft survival was monitored until rejection. (**B**) Survival graph for mice that received adoptively transferred cells derived from mice pretreated for 1 week with doxycycline. Doxycycline exposure continued in the recipient mice. (**C**) Survival graph for mice that received adoptively transferred cells derived from untreated mice. The cells were only exposed to doxycycline after adoptive transfer when it was added to the drinking water of recipient mice. Survival data in **B** and **C** were analyzed by log-rank test. The number of animals in each group is indicated. (**D**) Schematic of in vitro experimental plan. Control C57BL/6 WT Teffs (1 × 10^5^/well) (CD4^+^CD25^–^ responders [R]) were stimulated (S) with 1 × 10^5^/well of anti-CD3 and anti-CD28 beads or 2 × 10^4^ CBA (H-2^k^) DCs. CD4^+^CD25^+^ cells from shPhd2#9 or control mice were added at 1:1, 1:2, 1:4, and 1:8 ratios relative to Teff responders. Doxycycline at 1 μg/mL was added to all wells and replenished daily. Cells were incubated for 5 days, and ^3^H-thymidine was added for the final 18 hours of culture. Proliferation levels were normalized to the positive control (R + S). (**E**) In vitro suppression assay using anti-CD3 and anti-CD28 as stimulators. (**F**) In vitro suppression assay using DCs as stimulators. Data are represented as the mean ± SEM of biological replicates. Unpaired, independent groups (1:1, 1:2, 1:4, and 1:8) of 2 were analyzed by 2-tailed Student’s *t* test in **E** and **F**. *P < 0.05, **P < 0.01, and ****P < 0.0001.

Given the paradox of increased numbers of cells bearing Treg markers in mice with an autoimmune phenotype, we next tested the function of these cells. We assessed the ability of CD4^+^CD25^+^ cells to regulate the activity of effector cells in skin allograft rejection in vivo (Figure 7A). Immunodeficient C57BL/6 (H-2^b^) *Rag1^–/–^* mice were reconstituted with CD4^+^CD25^–^ effector T cells from WT C57BL/6 (H-2^b^) mice, either alone or in combination with shPhd2 or control CD4^+^CD25^+^ Tregs (expressing Foxp3 at >85% purity, using previously established cell doses [ref. 36]) and subsequently received a skin transplant from a CBA (H-2^k^) donor. shPhd2 CD4^+^CD25^+^ cells were isolated from mice carrying both *CAG-rtTA* and *TRE-Phd2* shRNA alleles, whereas control CD4^+^CD25^+^ cells were isolated from mice carrying the *CAG-rtTA* allele alone. In initial experiments, CD4^+^CD25^+^ cells were derived from donor animals pretreated with doxycycline to ensure that any change in function resulting from Phd2 KD had already developed (Figure 7B), and doxycycline treatment was continued for the duration of the experiment after transplantation to ensure that Phd2 KD was maintained. In these experiments, we observed that CD4^+^CD25^+^ cells from doxycycline-pretreated shRNA mice were unable to control skin rejection to the same degree that control CD4^+^CD25^+^ cells could.

In subsequent experiments, cells were obtained from mice of the same genotypes, but doxycycline treatment was only started after adoptive transfer into the recipient mice to ensure that the effects of Phd2 KD were being tested on cell subsets that were identical at the point of transfer into the C57BL/6 (H-2^b^) *Rag1^–/–^* recipients. Again, in contrast to the CD4^+^CD25^+^ cells from mice carrying the *CAG-rtTA* allele alone, we found that shPhd2 CD4^+^CD25^+^ cells were unable to control graft rejection (Figure 7C). In both assays, shPhd2 CD4^+^CD25^+^ cells provided no quantifiable benefit to graft survival, with grafts being rejected at least as quickly as was seen in mice receiving effector cells alone, indicating a total loss of regulatory function in this in vivo model.

In parallel, in vitro experiments were performed to assess the ability of doxycycline-treated CD4^+^CD25^+^ cells, from the same sources, to suppress the proliferation of CD4^+^CD25^–^responder cells stimulated polyclonally with anti-CD3 and anti-CD28 beads or with allogeneic DCs (Figure 7D). In both assays (Figure 7, E and F, respectively), CD4^+^CD25^+^ cells from doxycycline-treated shPhd2 animals were less effective at regulating the proliferative response than were cells from control animals. Taken together, the results of both in vivo and in vitro experiments indicate that CD4^+^CD25^+^ cells are hypofunctional as immune regulators when *Phd2* levels are knocked down, suggesting that deficiency of Treg function may contribute to the autoimmune phenotype we observed.

**Figure 8:**
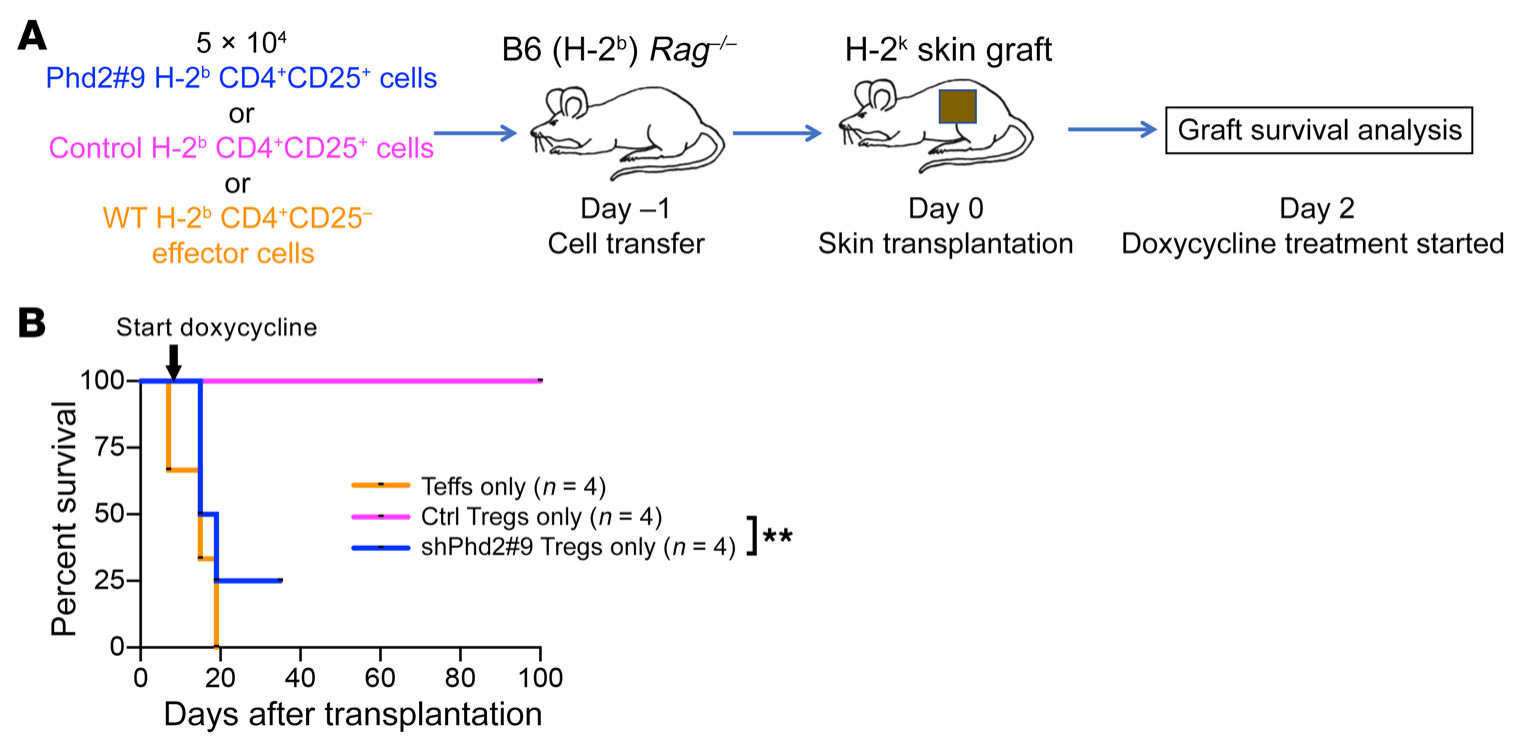
*Phd2*-KD CD4^+^CD25^+^ cells alone can induce skin allograft rejection. (**A**) Schematic of the in vivo experimental plan. C57BL/6 *Rag1^–/–^* (H-2^b^) mice received either 5 × 10^4^ H-2^b^ CD4^+^CD25^+^ cells from shPhd2#9 mice; 5 × 10^4^ H-2^b^ CD4^+^CD25^+^ cells from control mice; or 5 × 10^4^ H-2^b^ WT CD4^+^CD25^–^ effector cells alone. One day later, mice received an allogeneic H-2^k^ skin graft, followed by doxycycline treatment (2 mg/ml in the drinking water, ad libitum) from day 2 after transplantation. Allograft survival was monitored until rejection. (**B**) Skin allograft survival graph. Allograft survival data were analyzed by log-rank test. The censored data point in the shPhd2#9 group represents a mouse that developed weight loss and a generalized inflammatory phenotype. **P < 0.01, by log-rank test.

Noting that skin graft rejection appeared slightly faster in the presence of CD4^+^CD25^+^ shPhd2 cells than when effector cells were present alone, we tested the ability of CD4^+^CD25^+^ shPhd2 cells to reject a skin graft in isolation (Figure 8A). Intriguingly, cells selected with this surface phenotype prior to doxycycline treatment and then adoptively transferred into *Rag1^–/–^* recipients were able to reject skin allografts when these mice were treated with doxycycline, whereas we observed long-term graft survival in the doxycycline-treated recipients of CD4^+^CD25^+^ cells from control mice (Figure 8B). This indicates that Phd2 KD may lead to a converse effector function in this population of cells. Additionally, the shPhd2 recipient mice, but not those receiving control cells, developed features of the phenotype observed in shPhd2 mice, as these mice became unwell and lost weight, which led to early termination of the experiment for 1 animal. The loss of regulatory function and/or conversion to effector functionality can therefore induce the development of an autoimmune phenotype in shPhd2 mice.

**Figure 9:**
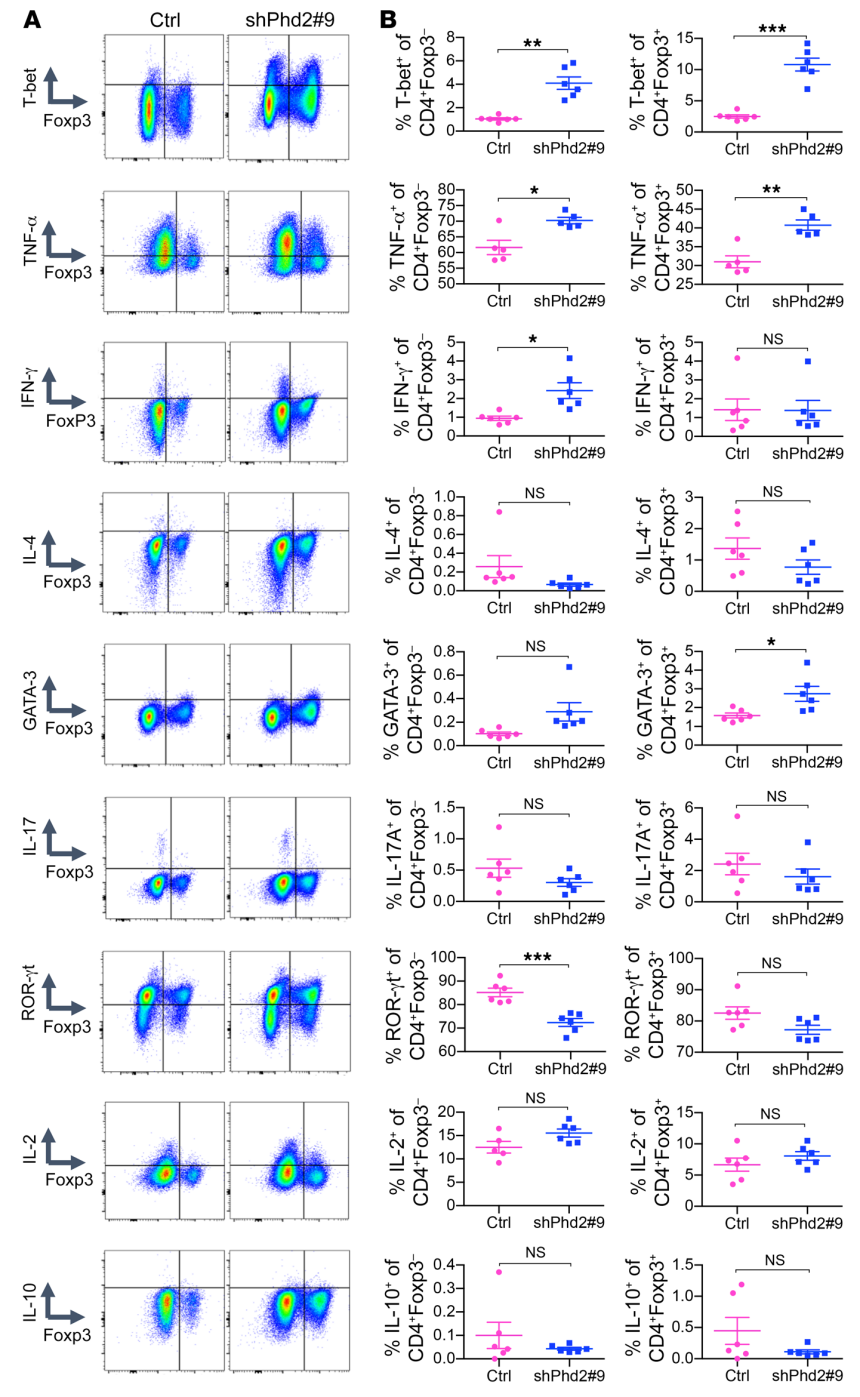
Expression patterns of T cell–related cytokines and transcription factors in shPhd2#9 mice. (**A**) Representative flow cytometric plots showing expression of T-bet, TNF-α, IFN-γ, IL-4, GATA-3, IL-17, ROR-γt, IL-2, and IL-10 against Foxp3 within the total CD4^+^ cell populations from pLNs of control and shPhd2#9 mice following 4 weeks of doxycycline treatment (2 mg/ml with 30% sucrose drinking water, ad libitum). (**B**) Percentage of cells expressing T-bet, TNF-α, IFN-γ, IL-4, GATA-3, IL-17, ROR-γt, IL-2, and IL-10 within CD4^+^Foxp3^–^ or CD4^+^FoxP3^+^ cell populations from pLNs of control and shPhd2#9 mice after 4 weeks of doxycycline treatment (*n* = 6 mice per group). Data are represented as the mean ± SEM. *P < 0.05, **P < 0.01, ***P < 0.001, by 2-tailed Student’s *t* test.

**Figure 10:**
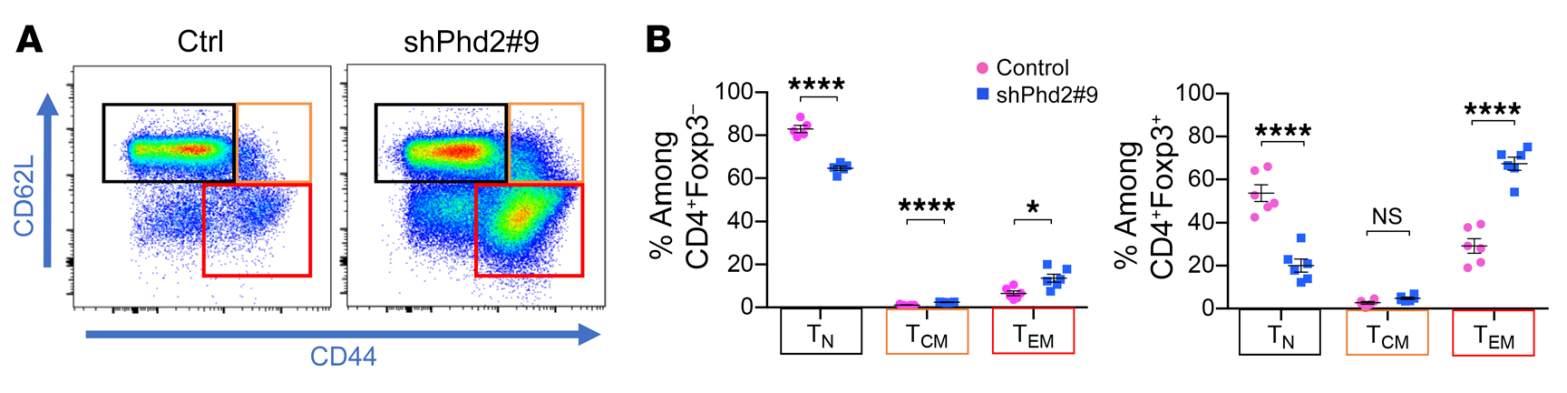
Identification of naive and memory T cells in shPhd2#9 mice. Dot plots and quantification of naive (T_N_) (CD44^lo^CD62L^hi^), central memory (T_CM_) (CD44^hi^CD62L^hi^) and effector memory (T_EM_) (CD44^hi^CD62L^lo^) T cells within pLNs of control and shPhd2#9 mice following 4 weeks of doxycycline treatment (2 mg/ml with 30% sucrose drinking water, ad libitum) (*n* = 6 mice per group). Data are represented as the mean ± SEM. *P < 0.05 and ****P < 0.0001, by 2-tailed Student’s *t* test.

To analyze the basis for these functional differences, we compared the expression of key transcription factors and cytokines in CD4^+^Foxp3^+^ and CD4^+^Foxp3^–^ cells from shPhd2 and control mice (Figure 9 and Supplemental Figure 7). We showed that despite ongoing expression of Foxp3, cells within this population from shPhd2 mice expressed increased levels of T-bet and TNF-α, but not IFN-γ, RORγt, GATA-3, IL-2, IL-4, IL-10, or IL-17. To take this analysis further, we examined the expression of CD44 and CD62L in CD4^+^Foxp3^+^ and CD4^+^Foxp3^–^ cells from shPhd2 and control mice and found that in both populations, Phd2kd led to a reduction in cells with a CD44^lo^CD62L^hi^ naive phenotype and an increase in CD44^hi^CD62L^lo^ effector memory cells (Figure 10 and Supplemental Figure 8).

## Discussion

Here, we report on a model for the genetic investigation of HIF/PHD hypoxia signaling pathways in the mouse as well as on the occurrence of a systemic autoimmune phenotype following suppression of the principal HIF prolyl hydroxylase PHD2. Following shRNA-mediated KD of *Phd2*, the mice developed a multilineage leukocyte expansion that was associated with destruction of lymphoid organ architecture, immune infiltration of major organs, ANAs. In keeping with the stochastic nature of immune responses, this phenotype was somewhat variable between individual KD mice, but overall, the phenotype was transferrable by BMT and reversible when doxycycline-driven, shRNA-mediated suppression of *Phd2* was stopped.

These findings strongly suggested the occurrence of an autoimmune syndrome but were somewhat surprising in view of early reports on general inactivation of *Phd2* using conditionally activated Cre recombinase, which did not highlight such a phenotype. Nevertheless, near-identical phenotypes obtained using 2 distinct shRNAs and complete suppression by coincident shRNA-mediated KD of *Hif2a* strongly suggested that the phenotype reflected on-target actions of the interventions. We therefore reexamined the phenotype of Cre recombinase–mediated *Phd2* inactivation in an additional set of mice. These experiments revealed a similar, though somewhat less severe, inflammatory phenotype associated with the development of ANAs, which was also suppressed by combined inactivation of *Phd2* with *Hif2a*. Though it is possible that this phenotype was obscured in the earlier studies by other, ultimately fatal, consequences of *Phd2* inactivation, we note that one of the earlier reports of *Phd2*-KO mice noted the occurrence of leukocytosis and thrombocytosis, such as we observed in the present study (14). Taken together, our findings indicate that *Phd2* KD or KO in adult mice has the potential to cause an autoimmune phenotype driven by cells originating within the BM.

It is unclear to us why this phenotype was generally stronger in the KD mice than in the KO animals, although we cannot exclude the possibility of a sensitizing effect of RNA interference or doxycycline. However, no such effects have been described in multiple reports of the use of the same doxycycline-inducible shRNA system to target other genes (37–40). Other important differences between the KD and KO systems could be relevant. In our experience, recombination between the loxP sites in the conditional Phd2-KO allele was always less than complete in any cell population within the *Rosa-CreER*–KO (Phd2-KO) mice that we used. Thus, it could be that residual, normal unrecombined cells were sufficient to significantly suppress the phenotype, whereas in the KD system, all cells would be expected to be affected by the shRNA expression, and thus no potentially compensatory normal cells would be present. It is equally possible that complete KO of *Phd2* in certain cells leads to their deletion and hence a phenotype that is somewhat less severe. Whatever the explanation for these quantitative differences, the occurrence of a qualitatively similar phenotype was reproducible across the 2 models and therefore potentially relevant to both the physiology of immune regulation and the clinical development of PHD2 and HIF2α inhibitors.

Tregs, which express the forkhead box transcription factor Foxp3, are key to immune homeostasis, and their disruption results in pathology with a number of similarities to those we observed in shPhd2 mice. Scurfy mice have a disabling mutation of Foxp3, which leads to a fatal lymphoproliferative disorder (41). Human mutations in the *FOXP3* gene cause an X-linked syndrome (IPEX) characterized by immune dysregulation, polyendocrinopathy, and enteropathy (42, 43). Although it is formally possible that the development of ANAs in our model was simply a consequence of the expansion of the total number of B cells, similarities in the lymphoid pathology of these conditions to that observed in shPhd2 mice led us to further examine Tregs (shPhd2 mouse cells bearing the Treg markers CD4^+^CD25^+^ and/or Foxp3^+^) from these mice. In contrast to most other models of immune dysregulation, we found that the absolute number of these Tregs in shPhd2 mice was increased compared with that in controls and that their ratio relative to the cell populations they control was also increased in the LNs but not the spleen. However, when equal numbers of cells were directly compared, the ability of Tregs (as defined by the expression of these markers) from shPhd2 animals to suppress either skin allograft rejection or in vitro responder T cell proliferation was impaired. The TSDR within the Foxp3 locus of shPhd2-derived CD4^+^CD25^+^ cells remained demethylated. Since expression of Foxp3 is dependent on selective demethylation of the TSDR (44), this finding is consistent with the maintained expression of Foxp3 we observed. Furthermore, the absence of epigenetic changes is consistent with the reversible nature of the phenotype upon doxycycline withdrawal in shPhd2 mice, which is relatively rapid in relation to the normal lifespan of Tregs. Phenotypically, these cells increased their expression of T-bet and TNF-α and gained a CD44^hi^CD62L^lo^ effector memory phenotype, but did not manifest features of Th17 conversion. Taken together, these results suggest that there is a functional switch downstream of, or independent from, Foxp3. However, this change was not simply a loss of regulatory function, since shPhd2 KD, induced after cells expressing Treg markers were transferred into an immunodeficient recipient, resulted in cells being present that acted positively to cause skin allograft rejection in the absence of any other effector cell population, indicating that a functional inversion had occurred within the transferred cell population.

Several studies have reported abnormalities in lymphocyte differentiation following genetic interventions on HIF or its regulatory machinery, in specific subsets of lymphocytes (16–31, 33). These phenotypes include abnormalities of Tregs in some studies, but the results were not always concordant and differ from our current findings. For instance, inactivation of *Vhl*, the gene encoding the ubiquitin E3 ligase that targets hydroxylated HIFα polypeptides for proteasomal destruction, using Cre recombinase restricted by a *Foxp3* promoter, resulted in a reduction of Foxp3 expression and an inflammatory phenotype that was dependent on HIF1α (29).

Our studies in the new model are the first to our knowledge to interrogate T cell function in the setting of general inactivation of *Phd2*, a difference that is probably important in view of the potential for crosstalk between subsets of lymphocytes. The observation of cells bearing classical Treg markers, which were apparently hypofunctional in immune suppression, or even stimulatory, is unusual. Nevertheless the results are consistent with emerging data on functional heterogeneity in Tregs. For instance, it is increasingly clear that Foxp3 alone does not ensure stability of the suppressive function of Tregs (45, 46). A number of additional factors are important for this function, including signaling through the T cell receptor (TCR) and CD25 (47, 48) and other signaling pathways, such as Nrp1 (33, 49), Foxo1/3a (50), and Eos (51). Furthermore, the multifunctional potential of Tregs is increasingly thought to be required physiologically for specific responses that are dependent on microenvironmental cues (46, 52–55). Environmental cues identified to date include the presence of TLR ligands or inflammatory cytokines such as IL-1β and IL-6 (56–58). Local tissue hypoxia is an established consequence of inflammation, and our results suggest a mechanism by which, in this setting, hypoxia may provide an additional cue for Tregs to modify their behavior and assist in the potentiation of required immune responses.

Inhibitors of PHD2 are now undergoing late-phase clinical trials for the treatment of anemia in patients with kidney disease (59). In this setting, very low intermittent dosing schedules are used in an attempt to specifically target erythropoietin production in the diseased kidneys and liver. This contrasts with our use of high-dose doxycycline and widely expressed transgene promoters to knock down or knock out PHD2, so that it is unlikely that exposures to the interventions are equivalent. In preliminary studies using short-term (≤72 hours) treatment of isolated cells, we did not observe consistent effects of prolyl hydroxylase inhibitors on human Tregs. Nevertheless, we believe that our findings are important. Interestingly, the antihypertensive agent hydralazine has inhibitory action against the HIF hydroxylases (60) and causes “drug-induced” systemic lupus erythematosus in a dose-dependent manner (61). Importantly, the syndrome we describe and hydralazine-induced lupus are both reversible. Thus, attempts to enhance immunity by PHD2 inhibition in specific settings might be a clinically credible strategy meriting assessment in future studies. Our finding that the autoimmune syndrome was suppressed by concurrent KD of *Hif2a* also suggests that the newly developed HIF2α antagonists, currently in clinical trials for the treatment of renal cancer (62), might be assessed in autoimmunity and transplantation. Overall, our newly developed model for reversible intervention on the HIF/PHD system and the findings we believe to be novel on the dysregulation of immune function should be of interest as this emerging area of medicine is explored.

## Methods

### Constructs and shRNAs.

miR30-based shRNA–targeting vectors were subcloned as previously described (35, 63). To identify potent shRNAs targeting murine Phd2, ten different 97-bp oligonucleotides predicted from shRNA design algorithms (35) were XhoI-EcoRI subcloned into the miR-30 cassette of the pGIPZ vector and screened by quantitative PCR (RT-PCR) for effects on mRNA levels in MEFs. Sequences were introduced into MEFs by lentiviral infection, and after 48 hours of doxycycline (doxycycline; doxycycline hyclate) treatment (2 μg/ml), target gene mRNA levels were measured and normalized against *Actb*, and the percentage of KD in transgenic cells was compared with control cells, which received an shRNA against Firefly luciferase (Supplemental Table 1). For KD of murine *Hif1a* and *Hif2a*, five shRNAs against each target were designed and subcloned, and their KD levels were tested as described above.

### ES cell targeting and generation of transgenic mice.

The targeting and screening strategy for ES cell production followed the method described previously (35, 63). Two potent shRNAs against murine *Phd2* (shPhd2#9 97-mer, 5′-TGCTGTTGACAGTGAGCGCCGCCACAAGGTACGCAATAACTAGTGAAGCCACAGATGTAGTTATTGCGTACCTTGTGGCGTTGCCTACTGCCTCGGA-3′ and shPhd2#3 97-mer, 5′-TGCTGTTGACAGTGAGCGCACGCAATAACTGTTTGGTATTTAGTGAAGCCACAGATGTAAATACCAAACAGTTATTGCGTATGCCTACTGCCTCGGA-3′) were subcloned into a cassette that links EGFP and shRNA expression downstream of a *TRE* and targeted by RMCE into a defined locus downstream of the *Col1a1* gene in KH2 ES cells, which also express the reverse transactivator *rtTA* from the *Gt(ROSA)26Sor* promoter (35, 63). Southern blotting demonstrated correct transgene insertion, and doxycycline-inducible KD of endogenous *Phd2* was confirmed by RT-PCR and immunoblot analysis. Germline transgenic mice were generated using the standard blastocyst injection method. MEFs were generated from 13.5-day-old embryos according to standard protocols. A similar approach was adopted for the creation of KD mice targeting *Hif1a* and *Hif2a* (shHif1 and shHif2 mice, respectively) using the most efficient shRNAs identified in our preliminary screening (*shHif1a#5* 97-mer, 5′-TGCTGTTGACAGTGAGCGAACGGGCCATATTCATGTCTATTAGTGAAGCCACAGATGTAATAGACATGAATATGGCCCGTGTGCCTACTGCCTCGGA-3′ and *shHif2a#3* 97-mer, 5′-TGCTGTTGACAGTGAGCGAACACTTGATGTGGAAACGTATTAGTGAAGCCACAGATGTAATACGTTTCCACATCAAGTGTGTGCCTACTGCCTCGGA-3′).

Mice were subsequently intercrossed with *CAG-rtTA* reverse transactivator mice (The Jackson Laboratory) to generate heterozygous double-transgenic (*CAG-rtTA^+/–^* *TRE-shRNA^+/–^*) mice. Mice were backcrossed more than 6 times with C57BL/6JOlaHsd WT mice (Envigo). *Phd2* single-KD mice carried alleles of *CAG-rtTA^+/–^ TRE-shPhd2#9^+/–^* (shPhd2#9) or *CAG-rtTA^+/–^* *TRE-shPhd2#3^+/–^* (shPhd2#3). Control mice for KD experiments carried a single allele of *CAG-rtTA* but no *TRE* allele (*CAG-rtTA^+/–^ TRE^–/–^*) (control). For double-KD mice bearing both *Phd2*- and *Hif1a*- or *Hif2a*-KD alleles, *CAG-rtTA^+/+^* *TRE-shPhd2#9^+/–^* mice were crossed with *CAG-rtTA^–/–^* *TRE-shHif1a#5^+/–^* or *CAG-rtTA^–/–^* *TRE-shHif2a#3^+/–^* mice to produce *CAG-rtTA^+/–^* *TRE-shPhd2#9^+^* *TRE-shHif1a#5^+^* (shPhd2Hif1) or *CAG-rtTA^+/–^* *TRE-shPhd2#9^+^* *TRE-shHif2a#3^+^* (shPhd2Hif2) mice. To induce shRNA expression, shRNA-bearing and control mice were provided ad libitum access to drinking water containing 2 mg/mL doxycycline with 30% sucrose from the age of 8 to 12 weeks. All of the following KO mouse lines were also bred in our facilities: *RosaERTCre* *Phd2^fl/fl^* (Phd2-KO), *RosaERTCre* *Phd2^fl/fl^* *HIf1a^fl/fl^* (Phd2Hif1-KO), *RosaERTCre* *Phd2^fl/fl^* *HIf2a^fl/fl^* (Phd2Hif2-KO), and *RosaERTCre–* (control) mice and have been described before (64). Tissues from KO and control mice were harvested 4 weeks after receiving tamoxifen treatment for 1 week by oral gavage. All KD and KO mice were housed in the Functional Genetics Facility of the Wellcome Trust Centre for Human Genetics (University of Oxford, Oxford, United Kingdom) in individually ventilated cages, with the only reported positives on health screening over the entire time course of these studies being for *Helicobacter* species (spp.), *Tritrichomonas* spp., and *Entamoeba* spp. Food and water were provided ad libitum, and the animals were maintained on a 12-hour light/12-hour dark cycle. After the preliminary experiments, phenotyping experiments were blinded and randomized as indicated, and no animals were excluded from the study.

### Characterization of transgenic shRNA mice.

Following fixation with fresh 10% neutral buffered formalin, GFP expression was assessed by bright-field imaging of tissues obtained from *CAG-rtTA*–driven Phd2-KD shRNA mice treated with doxycycline (2 mg/ml drinking water with 30% sucrose ad libitum) for the duration indicated in the figure legends. Images were acquired using an image scanner (ChemiDoc XRS+ System with ImageLab Software, Bio-Rad Laboratories).

### Hematological analysis.

Mice were culled, and blood obtained from the inferior vena cava or heart was collected into heparin-prefilled tubes. Blood samples were immediately analyzed using a Sysmex KX-21N Automated Hematology Analyzer.

### ANA tests.

Mouse serum samples were diluted 10 times and added to slides precoated with human epithelial HEp-2 cells (Fluorescent ANA Test System, Immuno Concepts). These were then stained with a goat anti–mouse IgG Alexa Fluor 488 secondary antibody (Invitrogen, Thermo Fisher Scientific), diluted 1:500 in PBS. Fluorescent images were acquired using a fluorescence stereomicroscope as described previously (65).

### Expression analysis.

RNA was extracted from several mouse tissues using a TRI Reagent Kit (MilliporeSigma) or an RNeasy Kit (QIAGEN). Purified total RNA (0.5–1.0 μg) was reverse transcribed using an RT-PCR kit (High Capacity cDNA Reverse Transcription Kit, Applied Biosystems, Thermo Fisher Scientific). mRNA expression was determined by RT-PCR (Platinum SYBR Green, Invitrogen, Thermo Fisher Scientific) using the primer pairs for *Phd2* (forward, GCCCAGTTTGCTGACATTGAAC and reverse, CCCTCACACCTTTCTCACCTGTTAG); *Hif1a* (forward, TGCTCATCAGTTGCCACTTCC and reverse, CCATCTGTGCCTTCATCTCATCTTC); *Hif2a/EPAS1* (forward, ACGGAGGTCTTCTATGAGTTGGC and reverse, GTTATCCATTTGCTGGTCGGC); *Bnip3* (forward, GACGAAGTAGCTCCAAGAGTTCTCA and reverse, CTATTTCAGCTCTGTTGGTATCTTGTG); *Phd3* (forward, TCAACTTCCTCCTGTCCCTCATC and reverse, GCGAACATAACCTGTCCCATTTC); *Actb* (forward, CTAGGCACCAGGGTGTGAT and reverse, TGCCAGATCTTCTCCATGTC); *Hprt* (forward, GTTGGATACAGGCCAGACTTT and reverse, CCACAGGACTAGAACTGC); and *Egfp* (forward, TGCTCAGGTAGTGGTTGTCG and reverse, AGAACGGCATCAAGGTGAAC). Values were analyzed as the relative quantification (RQ = 2^–ΔΔCt^) against the expression levels of the housekeeping gene *Hprt* or *Actb* by Step One Plus Real-Time PCR System (Applied Biosystems, Thermo Fisher Scientific).

### Western blotting, flow cytometry, and antibodies.

Western blotting was performed according to standard protocols in our laboratory (66). In brief, tissues were snap-frozen in liquid nitrogen and homogenized using a glass homogenizer on ice. Protein was extracted using a standard protein lysis buffer (10 mM Tris, pH 7.5, 1% SDS, 10% glycerol, 6.87 M urea) supplemented with a protease inhibitor cocktail (Complete, Roche Diagnostics) and quantified using a Pierce BCA Protein Assay Kit (Thermo Fisher Scientific). Proteins were separated on a polyacrylamide gel (Tris-glycine 8%–16% mini, NuSep Inc., Generon) and transferred onto a PVDF membrane (Immobilon-P, 0.45 μm, MilliporeSigma). Primary antibodies used for Western blot analysis included those against PHD2 (NB100-2219, Novus Biologicals); HIF1α (NB100-479, Novus Biologicals); HIF2α (NB100-122, Novus Biologicals); PHD3 (produced in-house) (66); and β-actin (ab49900, Abcam), followed by HRP-conjugated secondary antibodies. Conversion of the chemiluminescent substrate was measured on an image scanner (ChemiDoc XRS+ System with Image Lab Software, Bio-Rad Laboratories).

For FACS flow cytometric analysis, single-cell suspensions were prepared from pLNs (subiliac and axillary), spleen, and femoral BM in FACS buffer (PBS, pH 7.4, 1% BSA). Following RBC lysis, cells were stained with antibodies according to the manufacturer’s protocol. Cell preparations were stained with 7-AAD (eBioscience, Thermo Fisher Scientific), and only live cells were analyzed. After washing, cells were measured using a BD FACSCanto II (BD Biosciences) and analyzed using BD FACS DIVA and FlowJo software (TreeStar). For intracellular Foxp3 measurement, cells were fixed and permeabilized using a Foxp3 staining kit (eBioscience, Thermo Fisher Scientific) before staining for Foxp3, according to the manufacturer’s protocol. The following antibodies were used for flow cytometric analysis: CD3 (clone 145-2C11, eBioscience); CD3 (clone 17A2, ebioscience); CD4 (clone GK1.5, eBioscience); CD8 (clone 53-6.7, eBioscience); B220 (clone 30-H12, eBioscience); CD45 (clone 30-F11, eBioscience); CD19 (clone 9B1, eBioscience); Gr-1 (clone RB6-8C5, eBioscience); CD25 (clone PC61, eBioscience); CD25 (clone PC61, BD Biosciences); Foxp3 (clone 30-F11, eBioscience); Foxp3 (clone FJK-16s, eBioscience); CD62L (clone MEL-14; BD Biosciences); CD44 (clone IM7, BioLegend); IL-17A (clone ebio17B7, eBioscience); IL-4 (clone BVD6-24G2, eBioscience); IFN-γ (clone XMG1.2, eBioscience); IL-2 (clone JES6-5H4, eBioscience); TNF-α (clone MP6-XT22, eBioscience); IL-10 (clone JES5-16E3, eBioscience); GATA-3 (clone L50-823, BD Biosciences); T-bet (clone ebio4b10, eBioscience); and ROR-γt (clone AFKJS-9, eBioscience). The secretion of IL-17A, IL-4, IFN-γ, IL-2, TNF-α, and IL-10 was determined by flow cytometry after a 5-hour stimulation with 1 μg/mL ionomycin (catalog 56092-82-1, MilliporeSigma), 100 ng/mL PMA (catalog P1585, MilliporeSigma), and 5 μg/mL brefeldin A (catalog 420601, BioLegend). Gating strategies are shown in Supplemental Figure 4.

### Histology.

Organ samples were fixed in fresh 10% neutral buffered formalin (MilliporeSigma) overnight and further subjected to routine histological procedures for embedment in paraffin. Sections (4-μm-thick) from at least 2 or 3 different animals per group were placed on slides adjacent to each other to enable cross-comparison within a slide after H&E staining. Whole tissue sections were scanned at ×400 magnification using a NanoZoomer S210 digital slide scanner and reviewed using NDP.view2 software (both from Hamamatsu Photonics). Tissues were initially analyzed and scored in a blinded fashion by an independent mouse histopathologist; representative images were subsequently chosen to illustrate key histological findings.

### Cell purification for adoptive transfer.

CD4^+^CD25^+^ and CD4^+^CD25^–^ cells were isolated using the Miltenyi MACS system (Miltenyi Biotec) as previously described (45). Single-cell suspensions were prepared from LNs and spleens of 8- to 12-week-old shPhd2#9, control, and WT C57BL/6 mice, red cells were lysed, and cells were resuspended in PBS plus 1% FBS. Cells (2 × 10^8^) were then stained with rat anti-mouse antibodies against CD8 (clone YTS 169, 80 μg); B220 (clone RA3-6B2, 100 μg); MHC class II (clone TIB 120, 100 μg); and Mac-1 (M1-70, 100 μg) (antibodies produced and purified from their respective hybridomas in-house) followed by magnetic negative depletion using sheep anti–rat IgG–coated Dynabeads (Dynal Biotech, Thermo Fisher Scientific, 500 μl beads per 2 × 10^8^ cells). After magnetic separation, the remaining cells were stained with anti-CD25 phycoerythrin (PE) antibody (clone PC61.5, eBioscience; 5 μl/10^8^ of the initial cell number) and then with anti-PE microbeads (Miltenyi Biotec; 20 μl/10^7^ CD4^+^ cells). For selection of the CD4^+^CD25^+^ component, positive cells were isolated by magnetic separation using the MACS system (Miltenyi Biotec), and the CD4^+^CD25^–^ effector cells were obtained from the negative fraction in the cell separation column. On reanalysis, CD4^+^CD25^+^ cells expressed Foxp3 to a purity of more than 85%.

### BMT.

BM was harvested from 8- to 12-week-old shPhd2#9 or control donors (both CD45.2 and C57BL/6 backgrounds) by flushing tibiae and femurs with PBS (Gibco BRL, Thermo Fisher Scientific) supplemented with 2% BSA (MilliporeSigma). Single-cell suspensions, minus settled aggregates, were washed once with PBS and BSA through a 70-μm nylon cell strainer (Falcon). Treated recipient mice (CD45.1 and C57BL/6) received a split lethal dose of 4.5 plus 4.5 Gy total body irradiation (TBI) followed by intravenous injection of 5 × 10^6^ BM cells. Antibiotic prophylaxis with co-trimoxazole in the drinking water was provided to mice before and immediately after transplantation. Mice were monitored by serial flow cytometric analysis of the peripheral blood. Eight weeks after irradiation and transplantation, cohorts of mice were started on doxycycline treatment.

### Skin transplantation.

CBA (CBA, H-2^k^), C57BL/6 (B6, H-2^b^), and C57BL/6 *Rag 1^−/−^* (H-2^b^) mice were obtained from and housed in the Biomedical Services Unit of the John Radcliffe Hospital (Oxford, United Kingdom) in individually ventilated cages, with the only reported positives on health screening over the entire time course of these studies being for *Helicobacter* spp., occasional mouse norovirus, and *Tritrichomonas* spp. Food and water were provided ad libitum, and the animals were maintained on a 12-hour light/12-hour dark cycle. Mice were randomized and groups split across cages. All scoring was done in a blinded fashion, and no animals were excluded from the study. Sex-matched mice between 6 and 12 weeks of age at the time of the first experimental procedure were used in all experiments. T cell–deficient (C57BL/6 *Rag1^−/−^*) mice were reconstituted intravenously with 1 × 10^4^ CD4^+^CD25^–^ effector cells from C57BL/6 mice with or without 1 × 10^4^ to 5 × 10^4^ CD4^+^CD25^+^ cells from WT C57BL/6, shPhd2#9, or control animals. The day after reconstitution, the mice received a CBA skin graft under general anesthesia. Full-thickness tail skin was transplanted into graft beds on the flanks of recipient mice (67). Grafts were then monitored daily, and graft rejection was defined by complete destruction of the skin.

### In vitro suppression assays.

Sample processing, cell isolation, and in vitro T cell analyses were performed by the Transplantation Research Immunology Group following established laboratory protocols. Assay performance and data reporting conformed with the Minimal Information About T cell Assays (MIATA) guidelines (68) and the Minimum Information about Tregs (MITREG) guidelines (69). For in vitro suppression assays, splenocytes from shPhd2#9 or control C57BL/6 (H-2^b^) mice pretreated with doxycycline in the water for 4 weeks were used as a source of CD4^+^CD25^+^ cells. CD4^+^CD25^+^ cells and effector T cells (Teffs) (CD4^+^CD25^–^) were isolated using a Dynabeads Untouched Mouse CD4 Cells Kit (catalog 11415D, Invitrogen, Thermo Fisher Scientific) followed by CD25^+^ cell enrichment using an anti–CD25-PE antibody (clone PC61.5, eBioscience, Thermo Fisher Scientific) and anti-PE microbeads (catalog 130-048-801, Miltenyi Biotec). Cells were counted using trypan blue staining, a hemocytometer, and a microscope. Cells were cultured at 37°C and 5% CO_2_ in complete medium consisting of RPMI-1640 (MilliporeSigma) supplemented with 10% FBS, penicillin-streptomycin (MilliporeSigma), and l-glutamine (MilliporeSigma). WT C57BL/6 or control Teffs at 1 × 10^5^/well were stimulated with 1 × 10^5^/well anti-CD3/anti-CD28 Dynabeads (catalog 11456D, Invitrogen, Thermo Fisher Scientific). Syngeneic Tregs were added at 1:1 and 1:2 ratios (Tregs/Teffs), and further serial dilutions were performed. Doxycycline at 1 μg/ml was added to all wells and replenished every day. Cells were incubated for 5 days and ^3^H thymidine (PerkinElmer) added for the final 18 hours of culturing. For suppression assays using allogeneic DCs as stimulators, 2 × 10^4^ previously cryopreserved CBA (H-2^k^) DCs were added to each well instead of beads. Data were obtained as cpm using a BetaPlate reader (PerkinElmer) and recalculated as the division index. CBA DCs were generated from BM cultured for 6 days in complete RPMI with GM-CSF and IL-4 (both from Peprotech), with replacement of cytokines every second day. DCs were then cryopreserved in medium containing 45% FBS, 45% RPMI, and 10% DMSO (MilliporeSigma) and stored in vapor-phase liquid nitrogen until required.

### TSDR analysis.

Methylation at the TSDR was assessed in flow-sorted CD4^+^Foxp3^+^ and CD4^+^Foxp3^–^ cells from doxycycline-treated shPhd2#9 or control mice. Methylation analysis was conducted by EpigenDx by pyrosequencing of bisulphite-modified DNA purified from frozen cells. Four representative CpG residues of the mouse Foxp3 TSDR were analyzed using their ADS568-FS2 assay. The demethylation percentage on the active X chromosome was calculated, and data from female mice were adjusted to allow for complete methylation of the TSDR on the inactivated X chromosome.

### Statistics.

All statistical analyses were conducted using GraphPad Prism (GraphPad Software). A 1-way ANOVA was used for multigroup comparisons together with Tukey’s or Dunnett’s multiple comparisons post hoc tests. Groups with 2 independent variables were tested using a 2-way ANOVA with Sidak’s correction for multiple comparisons. A repeated-measures ANOVA was used to compare groups over time. Unpaired, 2-tailed Student’s *t* tests were used to assess 2 independent groups. Allograft survival data were analyzed using the log-rank test. *P* values of less than 0.05 were considered statistically significant.

### Study approval.

All mouse experiments were performed using protocols approved by the Committee on Animal Care and Ethical Review at the University of Oxford and in accordance with the UK Animals (Scientific Procedures) Act 1986, under PPL numbers 30-3050, 30-2966, P38BE32DE, and P8869535A.

## Author contributions

Experiments were designed by CWP, AY, FI, JH and PJR. LED provided reagents and expertise related to the development of the KD mice. MLC and AY designed and tested the shRNA sequences. AY, BD, and DB created the KD mice, with input from CWP and PJR. TB and XC supplied and genotyped the KO animals, with input from CWP and PJR. KB, TLC, and EC conducted the ANA assays in a blinded fashion on samples provided by AY and PSM. CLS, PSM, RG, ES, and HK contributed to the histopathological analyses. K. Kranc and HL performed parallel experiments in a separate animal facility that corroborated findings reported in this manuscript. AY, FI, JH, JM, MU, KM, and K. Kawai acquired and analyzed the flow cytometric data. Data were predominantly analyzed and interpreted by AY, FI, JH, CWP, and PJR, with contributions from the other authors. AY and FI performed statistical analyses. The manuscript was written by CWP, PJR, and FI with input from AY, JH, PSM, BD, and CLS and reviewed by all the authors. Figures were prepared by AY, PSM, and K. Kawai, with input from the other authors. The following authors made comparable contributions: AY initiated the experimental work and remained involved throughout; JH contributed essential immunological expertise in defining the immunological phenotype; AY is named first, because his contributions were made over a longer period than those of JH; joint senior authors PJR and CWP initiated the project; CWP managed the project throughout; and FI contributed essential immunological expertise.

## Supplementary Material

Supplemental data

## Version 1 06/04/2019

In-Press Preview

## Version 2 07/29/2019

Electronic publication

## Version 3 07/31/2019

Correction made to the final sentence of the abstract.

## Version 4 09/03/2019

Print issue publication
