# Supplementary material for: Systemic silencing of Phd2 causes reversible immune regulatory dysfunction
Source: J Clin Invest. 2019 Jul 29;129(9):3640–56. doi: 10.1172/JCI124099 (PMC6715380; doi:10.1172/JCI124099)
Supplement: Supplemental data [file jci-129-124099-s023.pdf]

	name	shRNA sequence (Coding Sequence, forward)	%KD in MEF	%KD in KH2 ES
Phd2	#1	TGCTGTTGACAGTGAGCGATCCGTCACGTTGATAACCCAATAGTGAAGCCACAGATGTATTGGGTTATCAACGTGACGGACTGCCTACTGCCTCG	80	
	#2	TGCTGTTGACAGTGAGCGCGCTTGCCAGACGAAATTTAAATAGTGAAGCCACAGATGTATTTAAATTTCGTCTGGCAAGCATGCCTACTGCCTCG	77	
	#3	TGCTGTTGACAGTGAGCGCACGCAATAACTGTTTGGTATTTAGTGAAGCCACAGATGTAAATACCAAACAGTTATTGCGTATGCCTACTGCCTCG	91	79
	#4	TGCTGTTGACAGTGAGCGCGGTGTGAGGGTTGAACTCAAGTAGTGAAGCCACAGATGTACTTGAGTTCAACCCTCACACCTTGCCTACTGCCTCG	81	
	#5	TGCTGTTGACAGTGAGCGCAGACTGGGACGCCAAGGTAAGTAGTGAAGCCACAGATGTACTTACCTTGGCGTCCCAGTCTTTGCCTACTGCCTCG	74	
	#6	TGCTGTTGACAGTGAGCGAAGCGAGCGAGAGCTAAAGTAATAGTGAAGCCACAGATGTATTACTTTAGCTCTCGCTCGCTCTGCCTACTGCCTCG	42	
	#7	TGCTGTTGACAGTGAGCGCTTGCTGACATTGAACCCAAATTAGTGAAGCCACAGATGTAATTTGGGTTCAATGTCAGCAAATGCCTACTGCCTCG	80	
	#8	TGCTGTTGACAGTGAGCGACCCAATTCAGTCAGCAAAGACTAGTGAAGCCACAGATGTAGTCTTTGCTGACTGAATTGGGCTGCCTACTGCCTCG	78	
	#9	TGCTGTTGACAGTGAGCGCCGCCACAAGGTACGCAATAACTAGTGAAGCCACAGATGTAGTTATTGCGTACCTTGTGGCGTTGCCTACTGCCTCG	95	90
	#10	TGCTGTTGACAGTGAGCGAAAGTACAGCCAGCATACGCCATAGTGAAGCCACAGATGTATGGCGTATGCTGGCTGTACTTCTGCCTACTGCCTCG	65	
Hif1a	#1	TGCTGTTGACAGTGAGCGCCTGATGGAAGCACTAGACAAATAGTGAAGCCACAGATGTATTTGTCTAGTGCTTCCATCAGATGCCTACTGCCTCG	93	86
	#2	TGCTGTTGACAGTGAGCGAAGCCAGCAAGTCCTTCTGATGTAGTGAAGCCACAGATGTACATCAGAAGGACTTGCTGGCTGTGCCTACTGCCTCG	78	
	#3	TGCTGTTGACAGTGAGCGCAGCGATATGGTCAATGTATTCTAGTGAAGCCACAGATGTAGAATACATTGACCATATCGCTATGCCTACTGCCTCG	80	
	#4	TGCTGTTGACAGTGAGCGCCTCCTTTACCTTCATCGGAAATAGTGAAGCCACAGATGTATTTCCGATGAAGGTAAAGGAGATGCCTACTGCCTCG	80	
	#5	TGCTGTTGACAGTGAGCGAACGGGCCATATTCATGTCTATTAGTGAAGCCACAGATGTAATAGACATGAATATGGCCCGTGTGCCTACTGCCTCG	95	91
Hif2a	#1	TGCTGTTGACAGTGAGCGCCGACAGAATCTTGGAACTGATTAGTGAAGCCACAGATGTAATCAGTTCCAAGATTCTGTCGGTTGCCTACTGCCTCG	89	94
	#2	TGCTGTTGACAGTGAGCGACGGGCAAGTGAGAGTCTACAATAGTGAAGCCACAGATGTATTGTAGACTCTCACTTGCCCGGTGCCTACTGCCTCG	41	
	#3	TGCTGTTGACAGTGAGCGAACACTTGATGTGGAAACGTATTAGTGAAGCCACAGATGTAATACGTTTCCACATCAAGTGTGTGCCTACTGCCTCG	93	96
	#4	TGCTGTTGACAGTGAGCGAACCGGGCAAGTGAGAGTCTACTAGTGAAGCCACAGATGTAGTAGACTCTCACTTGCCCGGTGTGCCTACTGCCTCG	88	
	#5	TGCTGTTGACAGTGAGCGATCTGCCTATGAGTTCTACCATTAGTGAAGCCACAGATGTAATGGTAGAACTCATAGGCAGAGTGCCTACTGCCTCG	66	

Supplementary Table 1. shRNA sequences and validation of target gene knockdown. Sequences were introduced by lentiviral infection into mouse embryonic fibroblasts (MEF) and recombinase-mediated cassette exchange (RCME) into KH2 ES cells. Following 48 hours of doxycycline treatment (2μg/mL) target gene mRNA levels were measured, normalized against *Beta-Actin* and % knockdown in transgenic cells was compared with control cells which received an shRNA against *Firefly-Luciferase*. Induction of HIF target gene mRNA included *Phd3* (x3.5 and x 4.9 fold) and *Bnip3* (x5.2 and x5.1 fold) by *Phd2#3* and *Phd2#9* sequences respectively. Data are represented as means from n=3.

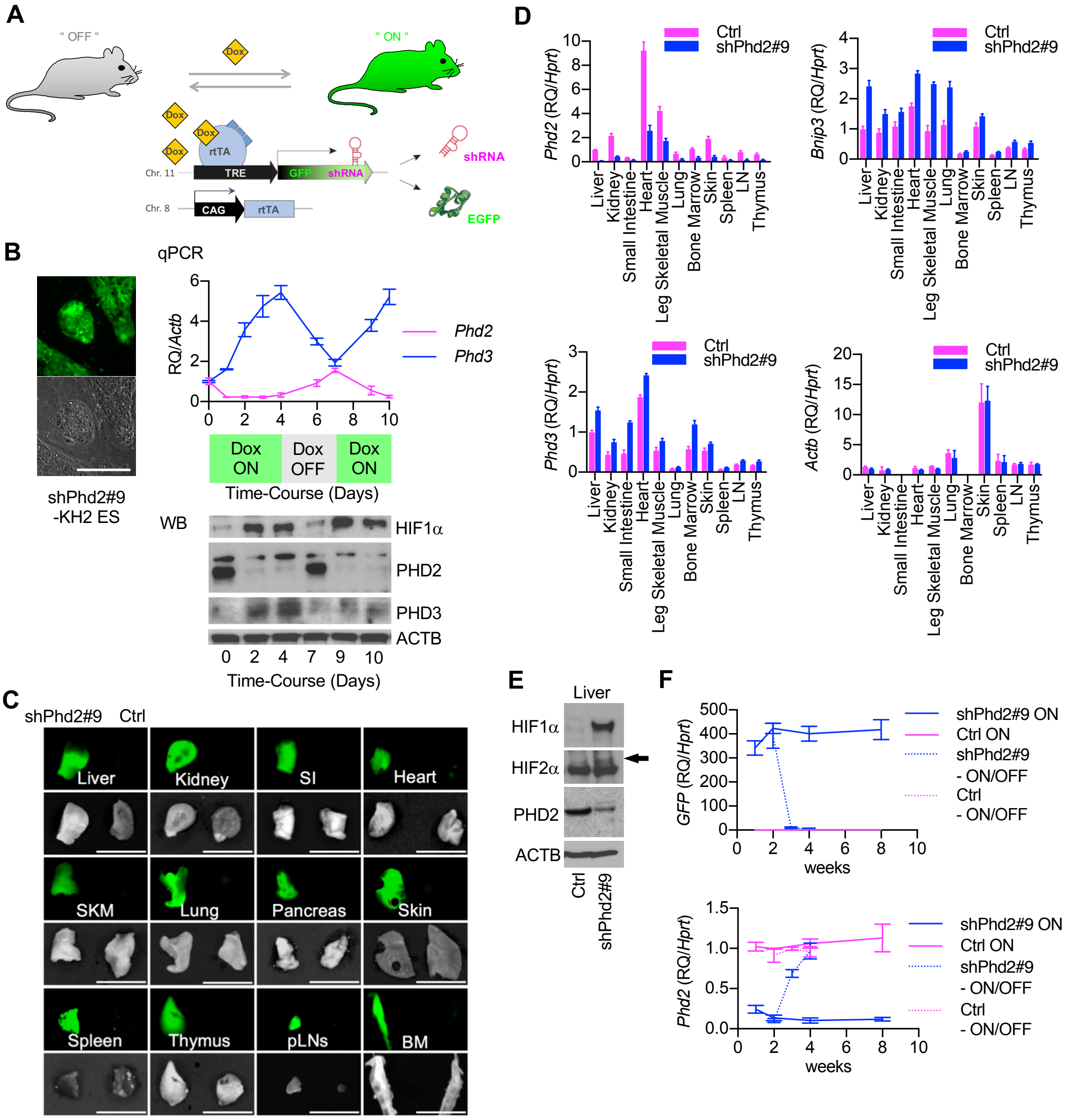

Supplementary Figure 1. Inducible shRNA *Phd2* knockdown mouse. (**A**) Schematic of *Phd2*-targeted shRNA transgenic strains, *CAG-rtTA;TRE-shPhd2*. These shPhd2 mice express a *CAG*-promoted tetracycline reverse transactivator (*rtTA*). In the presence of doxycycline (Dox) the transcription of an mRNA encoding *GFP* and a silencing shRNA directed against *Phd2* occurs. Control mice express the *CAG-rtTA* but not the shRNA/GFP cassette. (**B**) Representative GFP and brightfield images of shPhd2#9-KH2 ES cells (*R26-rtTA+/-;TRE-Phd2#9+/-*) after 2 days of doxycycline (1.0μg/mL in culture medium) treatment. Scale bar: 100μm. Time-course response of mRNA levels of the target gene (*Phd2*) and the downstream transcriptional effect of HIF-accumulation on *Phd3* mRNA in *shPhd2#9*-KH2 ES cells during Dox-ON and OFF treatment (n=3, values calculated relative to *Actb* (*bActin*) expression). Data are represented as means ± SD. The western blot (WB) shows the respective protein levels. (**C**) Representative GFP and brightfield images of organs harvested after 2 weeks of doxycycline treatment (2mg/mL with 30% sucrose drinking-water *ad libitum*) in shPhd2#9 and littermate control (Ctrl) mice. Peripheral lymph nodes (pLNs) and bone marrow (BM) following 4 weeks of doxycycline treatment. SI; Small Intestine, SKM; Leg Skeletal Muscle. Scale bar: 1cm. (**D**) Tissue distribution of *Phd2*, HIF target genes (*Phd3* and *Bnip3*) and non-HIF target genes (*Actb*) in organs harvested following two weeks of doxycycline treatment of shPhd2#9 mice and their littermate controls (Ctrl) (n=3, values calculated relative to *Hprt* expression as the relative quantity (RQ) compared with the liver from Ctrl mice). Data are represented as means ± SD. (**E**) Western blot data of HIF1α, HIF2α (arrow), PHD2, and loading control ACTB in the liver harvested following two weeks of doxycycline treatment of shPhd2#9 mice and their littermate controls (Ctrl). (**F**) Reversibility of changes to *GFP* and *Phd2* mRNA levels in doxycycline treated shPhd2#9 (Blue) mice and their littermate controls (Ctrl, Pink) in the liver. Dox-ON group (8 weeks of doxycycline treatment, Solid lines). Dox-ON/OFF group (2 weeks of doxycycline treatment followed by 2 weeks without doxycycline treatment, Dotted lines). N=3 at each time point in both groups, values calculated relative to *Hprt* expression. Data are represented as means ± SD.

A

	shPhd2#3	Ctrl
x4pLN (mg)	75; 90	8
Spleen (mg)	320; 280	92

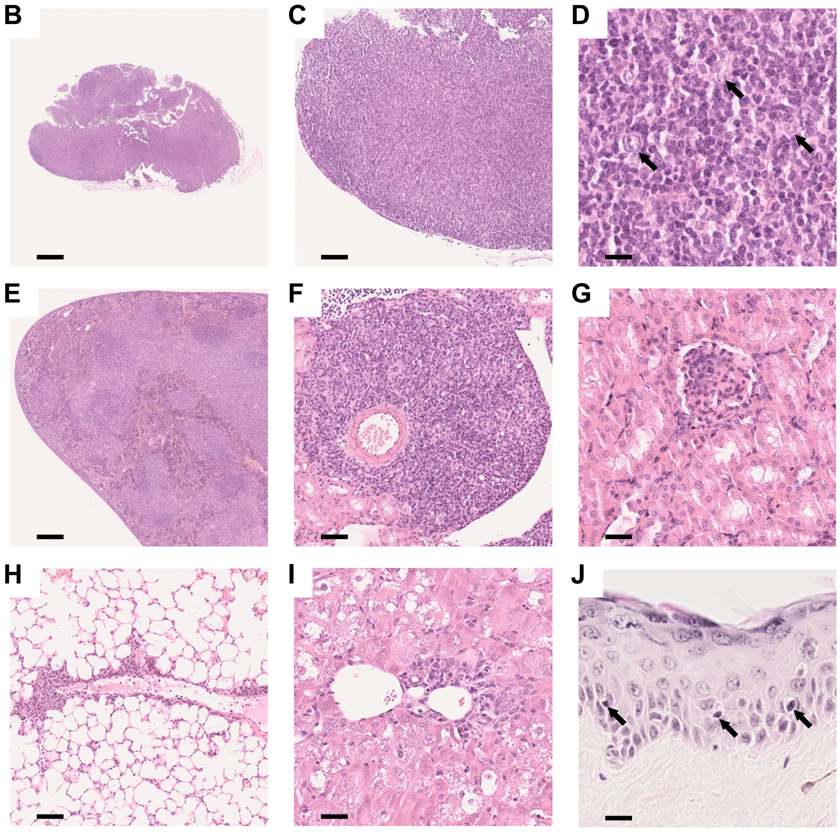

Supplementary Figure 2. Similar phenotypes develop in mice bearing a different shRNA sequence knocking down *Phd2*, shPhd2#3. (**A**) Tissue weight of peripheral lymph nodes (pLNs) (x4) and spleens from shPhd2#3 (*Phd2* knockdown) (n=2) and littermate control (Ctrl, n=1) mice treated with doxycycline in the drinking water (2mg/mL with 30% sucrose drinking-water *ad libitum*) for 8 weeks. H&E staining of tissues from shPhd2#3 mice: pLNs (x2.5 (**B**), x10 (**C**) and x40 (**D**)), arrows indicate cells with oval, vesicular nuclei and eosinophilic cytoplasm; spleen (x5 (**E**)); kidney (x20 (**F**) and x40 (**G**)); lung (x20 (**H**)); liver (x40 (**I**)); skin (x80 (**J**), arrows demonstrate exocytosis of lymphocytes into the epidermis). The scale bar is 500μm at x2.5 and proportionately smaller lengths at higher magnifications. The same experiment was repeated twice independently.

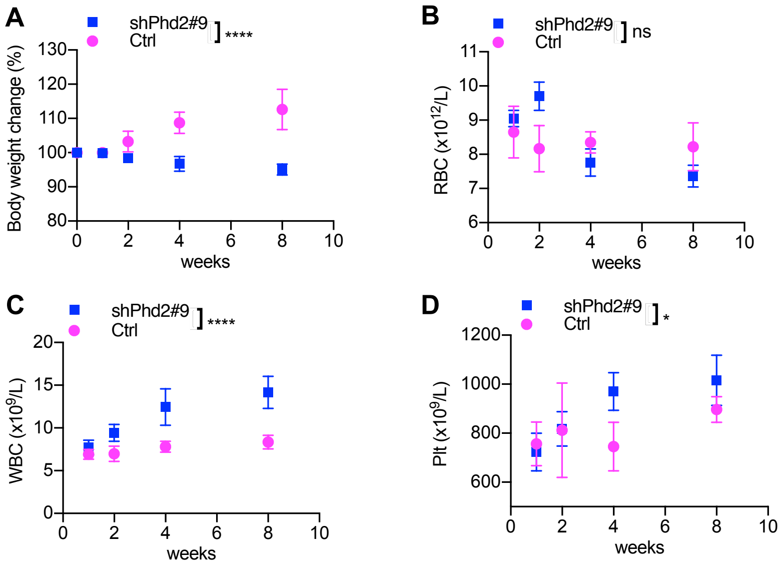

Supplementary Figure 3. Time course of changes in body weight and blood count differential after *Phd2* knockdown. (**A**) Body weight (**B**) red blood cell (RBC) counts, (**C**) white blood cell (WBC) counts and (**D**) platelet (Plt) counts from shPhd2#9 (Phd2 knockdown) and littermate control (Ctrl) mouse blood. Mice were treated for 8 weeks with doxycycline (2mg/mL with 30% sucrose drinking-water *ad libitum*). Data are represented as means ± SD, with n=3-8 per group at each time point gathered in two independent experiments. Groups were compared using a two-way ANOVA.

*p<0.05; ****p<0.0001; ns=not significant.

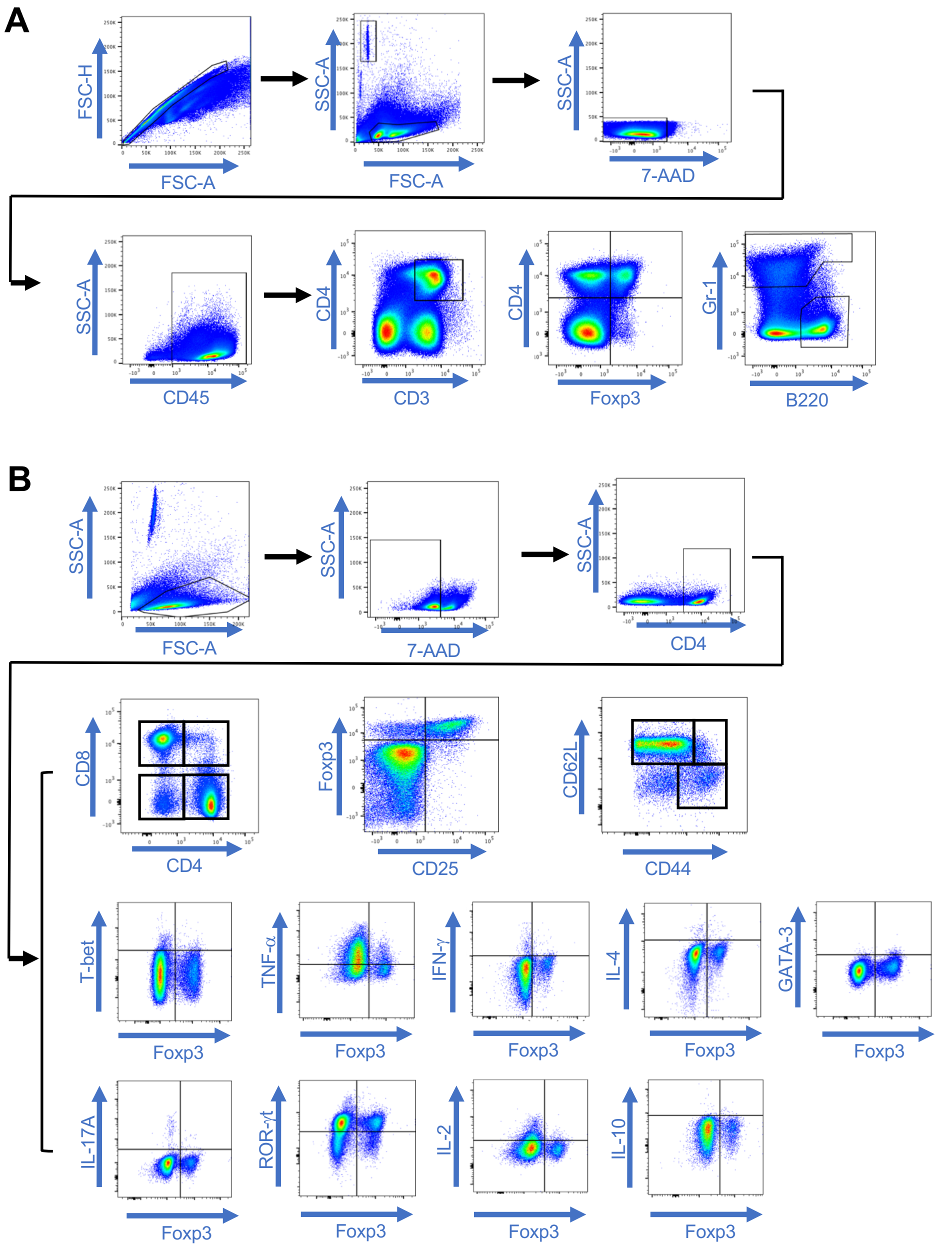

Supplementary Figure 4. Gating strategy for FACS analysis. Representative flow cytometry dot plots indicating the gating strategy **(A)** for identification of myeloid and lymphocyte populations in Figures 2, 4, and 5, and **(B)** for CD4^{+} lymphocyte subsets in Figures 6, 9 and 10. Control flow plots in Figure 6A, 6B, 9A, and 10 are reshown.

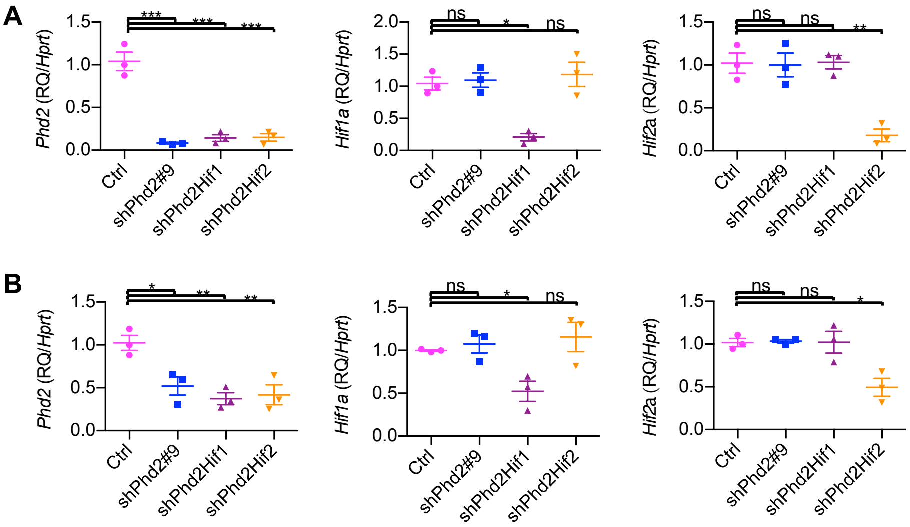

Supplementary Figure 5. *Phd2*, *Hif1a*, and *Hif2a* silencing in doxycycline-induced knockdown mice. mRNA levels of *Phd2*, *Hif1a*, *Hif2a* in the (**A**) liver and (**B**) spleen, harvested from shPhd2#9 (*Phd2* knockdown), shPhd2Hif1 (*Phd2* knockdown/*Hif1a* knockdown), shPhd2Hif2 (*Phd2* knockdown/*Hif2a* knockdown) and control (Ctrl) mice treated for 4 weeks with doxycycline (2mg/mL with 30% sucrose drinking-water *ad libitum,* RQ values against tissues from Ctrl mice). Data are represented as means ± SEM with n=3 per group. Multigroup comparisons were analyzed by one-way ANOVA with Tukey's multiple comparisons post hoc test.

*p<0.05; **p<0.01; ***p<0.001; ns=not significant.

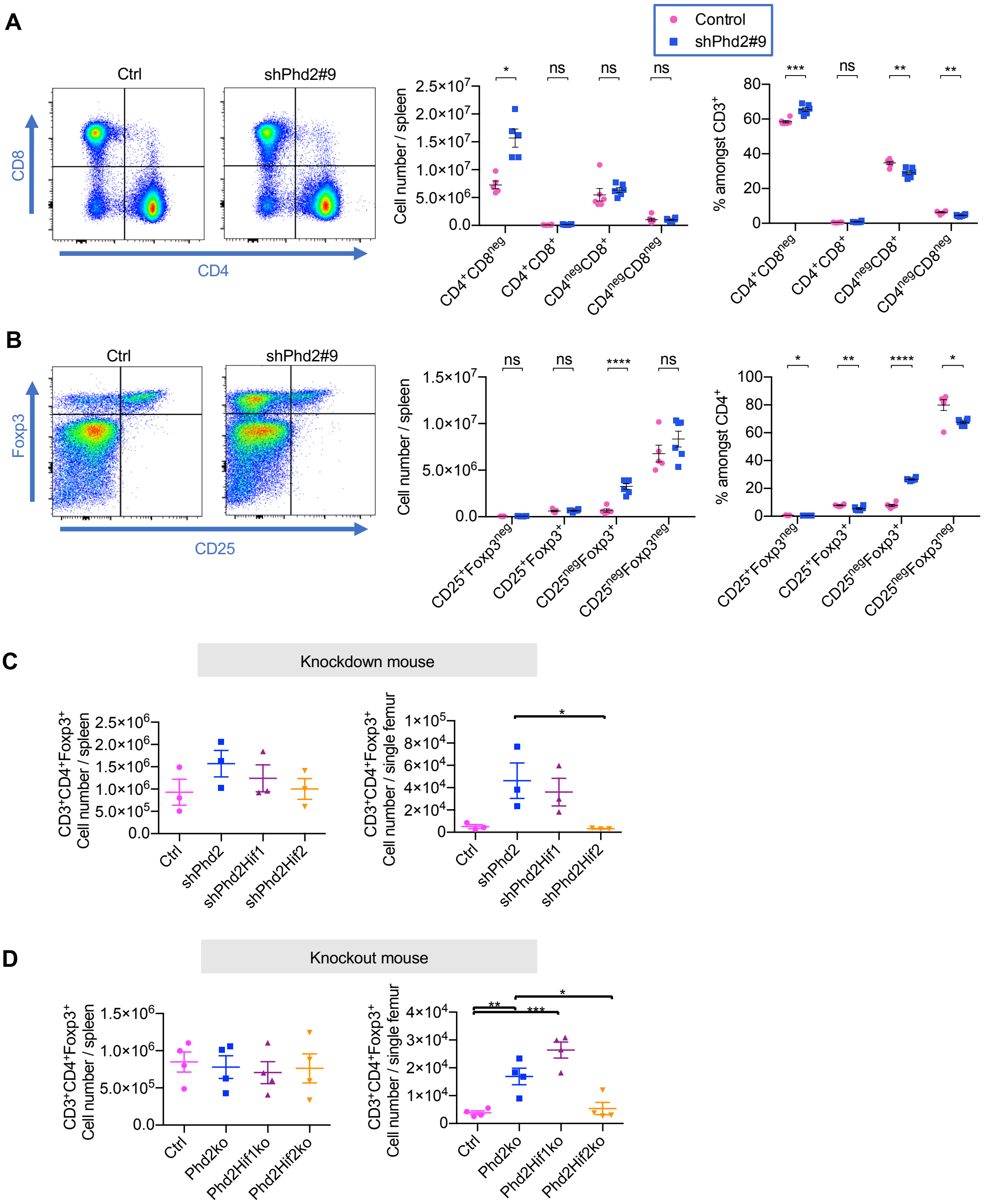

Supplementary Figure 6. Enumeration of cells bearing helper, effector and regulatory markers in *Phd2* knockdown and knockout mice. (**A**) Gating strategy of single positive ($CD4^+CD8^-$ or $CD4^-CD8^+$), double positive ($CD4^+CD8^+$) and double negative ($CD8^-CD4^-$) cells within spleen of control (Ctrl) and shPhd2#9 mice following 4 weeks of doxycycline treatment (2mg/mL with 30% sucrose drinking-water *ad libitum*). Absolute counts of each population per spleen (left) and expressed as a percentage of $CD3^+$ cells are shown. (**B**) Expression of Foxp3 and CD25 within $CD4^+$ populations within spleen of control (Ctrl) and shPhd2#9 mice following 4 weeks of doxycycline treatment. Representative dot plots and quantitation both as absolute counts per spleen and percentage amongst total $CD4^+$ cells are shown (**C**) $CD3^+CD4^+Foxp3^+$ cell numbers in spleen and bone marrow (femur) from shPhd2#9 (*Phd2* knockdown), shPhd2Hif1 (*Phd2* knockdown/*Hif1a* knockdown), shPhd2Hif2 (*Phd2* knockdown/*Hif2a* knockdown) and Ctrl mice. (**D**) $CD3^+CD4^+Foxp3^+$ cell numbers in spleen and bone marrow (Femur) from Phd2ko (*Phd2* knockout), Phd2Hif1ko (*Phd2* knockout/*Hif1a* knockout), Phd2Hif2ko (*Phd2* knockout/*Hif2a* knockout)and Ctrl mice. Data are represented as means ± SEM with at least n=3 per group. Unpaired, independent groups of 2 were analyzed using a 2-tailed Student's *t* test. Multigroup comparisons were analyzed by one-way ANOVA with Tukey's or Dunnett's multiple comparisons post hoc test.

*p<0.05; **p<0.01; ***p<0.001.

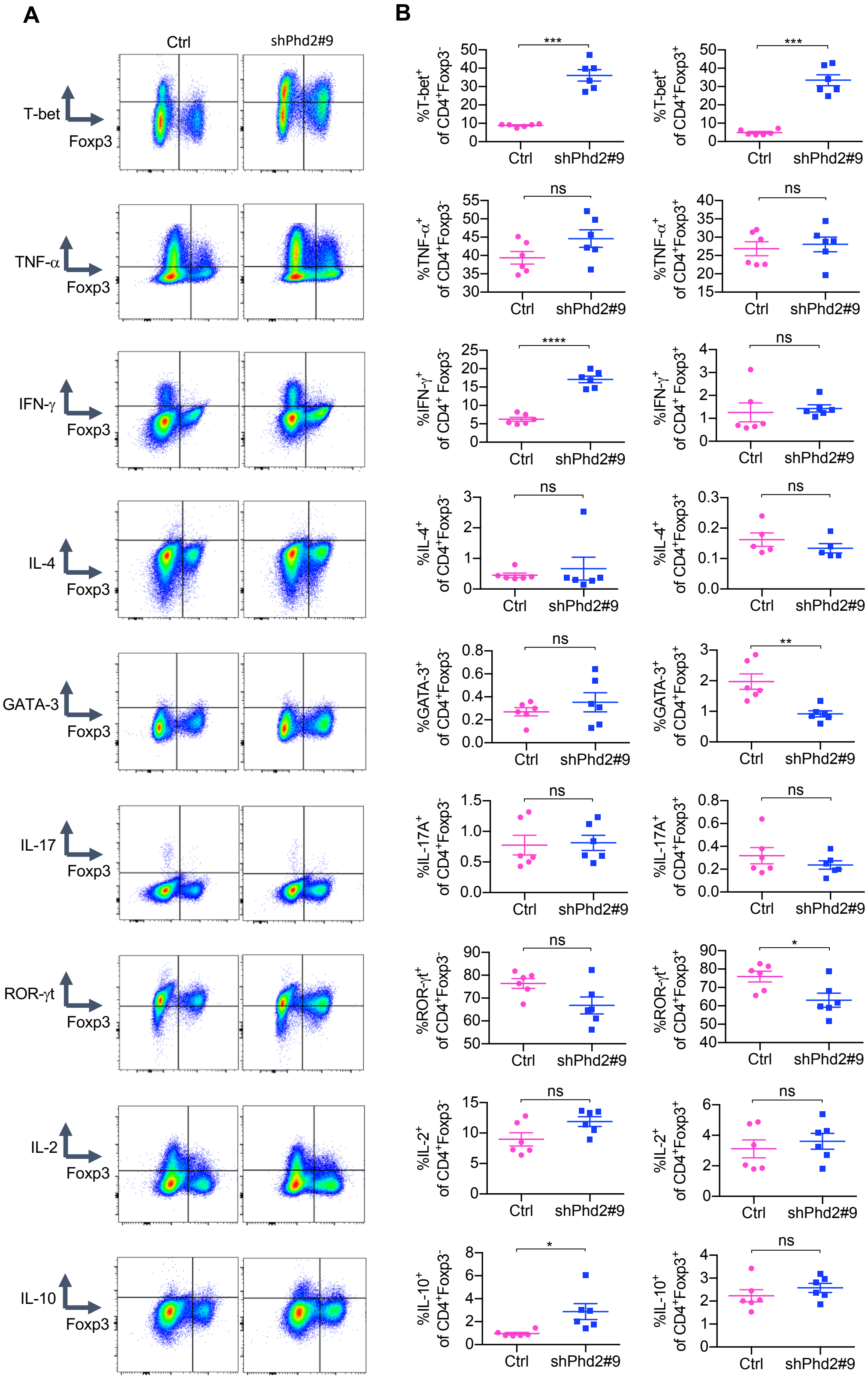

Supplementary Figure 7. Expression patterns of T cell related cytokines and transcription factors in shPhd2#9 mice. (**A**) Representative flow cytometry plots demonstrating expression of T-bet, TNF-α, IFN-γ, IL-4, GATA-3, IL-17, ROR-γt, IL-2 and IL-10 against Foxp3 within the total CD4+ populations from spleen of control (Ctrl, left) and shPhd2#9 mice (right) following 4 weeks of doxycycline treatment (2mg/ml with 30% sucrose drinking-water *ad libitum*). (**B**) Percentage of cells expressing T-bet, TNF-α, IFN-γ, IL-4, GATA-3, IL-17, ROR-γt, IL-2 and IL-10 within CD4+Foxp3- (left) or CD4+FoxP3+ cell populations (right) from spleens of Ctrl and shPhd2#9 mice after 4 weeks of doxycycline treatment (n = 6 mice per group). Data are represented as means ± SEM, analyzed using 2-tailed Student's *t* tests.

**p*<0.05; ***p*<0.01; ****p*<0.001; *****p*<0.0001; ns=not significant

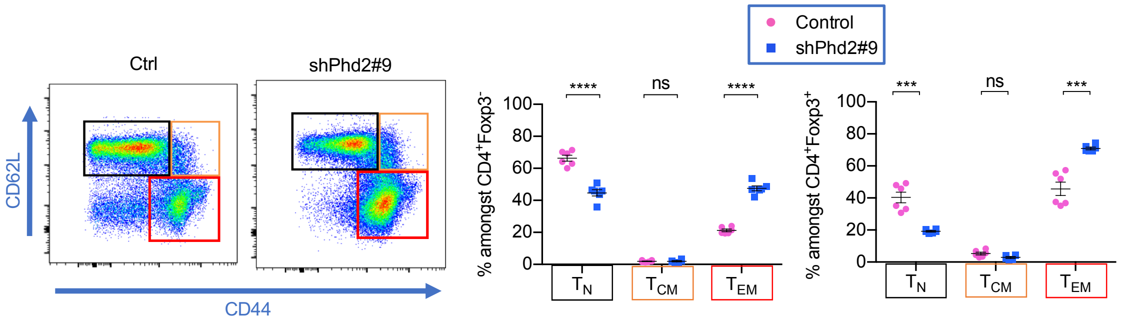

Supplementary Figure 8. Identification of naïve and memory T cells in shPhd2#9 mice. Dot plots and quantification of naïve (T_N, CD44^{lo}CD62L^{hi}), central memory (T_{CM}, CD44^{hi}CD62L^{hi}) and effector memory (T_{EM}, CD44^{hi}CD62L^{lo}) T cells within spleens of control (Ctrl, left) and shPhd2#9 mice (right) following 4 weeks of doxycycline treatment (2mg/ml with 30% sucrose drinking-water *ad libitum*) (n = 6 mice per group). Data are represented as means ± SEM, analyzed using 2-tailed Student's *t*-tests.

**p<0.01; ***p<0.001; ****p<0.0001; ns=not significant.
